# An early Transcriptomic Investigation in Adult Patients with Spinal Muscular Atrophy Under Treatment with Nusinersen

**DOI:** 10.1007/s12031-024-02251-1

**Published:** 2024-09-26

**Authors:** Maria Liguori, Annalisa Bianco, Alessandro Introna, Arianna Consiglio, Giammarco Milella, Elena Abbatangelo, Eustachio D’Errico, Flavio Licciulli, Giorgio Grillo, Isabella Laura Simone

**Affiliations:** 1 National Research Council, Department of Biomedicine, Institute of Biomedical Technologies - Bari Unit, 70125 Bari, Italy; 2 Neurology Unit, Department of Translational Biomedicine and Neuroscience, University of Bari “Aldo Moro”, 70124 Bari, Italy; 3 School of Medicine, University of Bari “Aldo Moro”, 70124 Bari, Italy

**Keywords:** Spinal muscular atrophy (SMA), miRNAs, mRNAs, HT-NGS, *SMN1*, *SMN2*, Nusinersen

## Abstract

Spinal muscular atrophy (SMA) is a rare degenerative disorder with loss of motor neurons caused by mutations in the *SMN1* gene. Nusinersen, an antisense oligonucleotide, was approved for SMA treatment to compensate the deficit of the encoded protein SMN by modulating the pre–mRNA splicing of *SMN2*, the centromeric homologous of *SMN1*, thus inducing the production of a greater amount of biologically active protein. Here, we reported a 10-month transcriptomics investigation in 10 adult SMA who received nusinersen to search for early genetic markers for clinical monitoring. By comparing their profiles with age-matched healthy controls (HC), we also analyzed the changes in miRNA/mRNAs expression and miRNA-target gene interactions possibly associated with SMA. A multidisciplinary approach of HT-NGS followed by bioinformatics/biostatistics analysis was applied. Within the study interval, those SMA patients who showed some clinical improvements were characterized by having the *SMN2/SMN1* ratio slightly increased over the time, while in the stable ones the ratio decreased, suggesting that the estimation of *SMN2/SMN1* expression may be an early indicator of nusinersen efficacy**.** On the other hand, the expression of 38/147 genes/genetic regions DE at T0 between SMA and HC like *TRADD* and *JUND* resulted “restored” at T10. We also confirmed the dysregulation of miR-146a(-5p), miR-324-5p and miR-423-5p in SMA subjects. Of interest, miR-146a-5p targeted *SMN1*, in line with experimental evidence showing the key role of astrocyte-produced miR-146a in SMA motor neuron loss. Molecular pathways such as NOTCH, NF-kappa B, and Toll-like receptor signalings seem to be involved in the SMA pathogenesis.

**Supplementary Information:**

The online version contains supplementary material available at 10.1007/s12031-024-02251-1.

## Introduction

Spinal muscular atrophy (SMA) is a rare autosomal-recessive degenerative disorder (incidence 1/10,000 live births) characterized by progressive loss of lower motor neurons with subsequent progressive muscle weakness and, in the most severe forms, early death due to respiratory failure (Ahmad et al. 2016; Jha et al. 2018). A wide range of SMA phenotypes has been described according to age of onset and level of motor functions achieved, ranging from early onset in very weak infants unable to sit unsupported, to adult onset SMA with slow rate of progression (type 1–4) (Lunn and Wang 2008; Wang and Lunn 2008; Wirth et al. 2020). Pathogenetically, the disease is caused by mutations in survival motor neuron-1 (*SMN1*) gene (locus 5q11.2-q13.3), which lead to the loss of functions of the encoded protein SMN. However, up to 10% of full-length SMN is produced by the centromeric homologous *SMN2*, which partially compensates for the insufficient product of mutated *SMN1. SMN2* copy number seems to correlate with the disease severity, contributing to the reported SMA clinical heterogeneity (Yang et al. 2016). SMN is an almost ubiquitously expressed protein so the vulnerability of motor neurons to its quantity/quality defect is not completely clear. Many concurrent causes have been evoked, e.g., the impact of the surrounding glial cells (mainly astrocytes) on the neurons functions (at different levels), as well as the involvement of other modulating genes or modifiers factors such as microRNAs (miRNAs) or long non-coding RNA (lncRNAs) (Tu et al. 2017). Indeed, SMN seems to be involved in RNA metabolism, especially in small nuclear ribonucleoprotein (snRNP) biogenesis, alternative splicing, trafficking of RNA-binding proteins and translation of target mRNAs in neurites (Gubitz et al. 2004; Fallini et al. 2014).

Between 2016 and 2017, an innovative drug for treating SMA was approved in several Countries including Italy. Nusinersen is an antisense oligonucleotide (ASO) developed by Ionis Pharmaceuticals and Biogen that modulates the pre–mRNA splicing of the *SMN2* gene, thus inducing the production of a greater amount of full-length, biologically active SMN. Since nusinersen cannot cross the blood–brain barrier, it is administered intrathecally. In a mouse model of SMA, nusinersen showed to ameliorate the motor neuron vitality, leading to improvement in muscle physiology, motor function, and cell survival (Passini et al. 2011). In human clinical trials, nusinersen demonstrated its effectiveness in infants and children affected by SMA (Finkel et al. 2017; Mercuri et al. 2018; Darras et al. 2019). No clinical trials were performed in adult SMA; however, the real-world data on its safety and, although more limited, efficacy also in SMA type 2–3-4 supported the extensive use of nusinersen in all SMA subtypes (Maggi et al. 2020; Arslan et al. 2023).

From the biochemical point of view, evidence showed significant decreasing levels of markers of neurodegeneration like neurofilaments (NFL) in CSF of infantile-onset SMA during nusinersen treatment, suggesting a recovery in motor neuron damage, whereas in CSF and serum of adolescent and adult SMA it fails to act as a reliable marker of treatment response (Olsson et al. 2019; Winter et al. 2019). Recently, the role of amyloid β-peptide as another potential pharmacodynamic biomarker in SMA has been explored, although without reaching a final consensus due to opposite results (Walter et al. 2019; Introna et al. 2021).

So far, little information has been reported on the impact of nusinersen on the other hypothesized players in the pathogenesis of SMA, e.g., the circulating miRNome (Catapano et al. 2016), especially in adult SMA patients. MiRNAs are endogenous small non-protein-coding RNA molecules (~ 22 nucleotides) that post‐transcriptionally regulate the gene expression across multiple tissues. Several tissue-specific miRNAs, such as miR-1, miR-9, miR-132, miR-206, miR-183, miR-375, and miR-133a/b, have been proposed as reliable biomarkers of SMA course and prognosis (Magen et al. 2022; Abiusi et al. 2021), as well as promising biomarkers for monitoring the response to nusinersen in SMA type-1 patients (Zaharieva et al. 2022).

To shed further light on the transcriptomic (miRNA/mRNA) impact of this quite novel treatment, we performed a longitudinal observation (10 months) on a cohort of adult SMA who received nusinersen as the first disease-modifying therapy. The primary goal was to search for suitable molecular markers (miRNAs) for early treatment monitoring and responsiveness that may be used in clinical practice.

As second goal, by comparing the transcriptomic profiles of adult SMA patients naïve for any treatment (T0) and age-matched healthy controls (HC), we searched for possible changes in the mRNA expression and miRNA-target gene interactions to add further information in the overall pathogenetic mechanisms of the disease. For both these aims, we took advantage from an experienced multidisciplinary approach applied in in other complex neurological diseases (Liguori et al. 2018a, b).

## Materials and Methods

### Subjects’ Recruitment and Clinical Evaluation

Consecutive adult patients with SMA type 2–3-4 were recruited among those diagnosed at the Neurology Unit of the Department of Translational Biomedicine and Neuroscience, University of Bari (Bari, Italy). For the diagnosis of SMA, published criteria (Lunn and Wang 2008) were satisfied and confirmed by the genetic tests identifying 5q SMA homozygous gene deletion, homozygous mutation, or compound heterozygote. At T0 (before first dose) and after 10 months of nusinersen treatment (T10), SMA patients were evaluated by experienced neurologists, and their clinical disabilities were measured by using Revised Upper Limb Module (RULM) and Hammersmith Functional Motor Scale (HFMS) (Mazzone et al. 2014, 2017). Patients who fulfilled the below mentioned inclusion criteria were informed about the protocol and the drug (see below). In case of written approval, they were treated with nusinersen administered via lumbar puncture according to therapeutic protocol in hospital settings.

#### Inclusion Criteria

For the administration of nusinersen, given the complexity of the procedure, patients were informed about the lumbar puncture and all the possible obstacles that may derive from spinal or other anatomical abnormalities. Written informed consent was signed by each patient. Recruited patients were subjects over 18 years of age with SMA diagnosis type 2–3 genetically documented and number of *SMN2* copies ≤ 4. They also had the ability to understand and comply with the study, as well as the ability to give written informed consent.

#### Exclusion Criteria

Patients with other motor neuron diseases than genetic SMA were excluded, as well as those complaining preexisting conditions such as HIV, clinically significant chronic hepatitis, or other active infections. For the therapy administration, all the clinical conditions that obstructed the intrathecal puncture, or the abnormalities of coagulation that could result in complications during the surgical procedure, were considered exclusion criteria. Also, female patients with verified pregnancy (by laboratory testing) or lactating were not considered candidates for the therapy.

#### Treated SMA Group

Nusinersen was intrathecally administered according to standard protocol (https://www.ema.europa.eu/en/medicines/human/EPAR/spinraza) in hospital setting. As well as the clinical observation of each patient, their transcriptomic profiles were analyzed at T0 (before the first dose) and after 10 months.

The sample size was chosen to ensure a theoretical statistical power greater than 80% with a false discovery rate ≤ 0.05 in comparing RNA expression between conditions. All parameters were set based on the results obtained in previous studies with similar experimental conditions (Liguori et al. 2018a, b). With the proposed experimental conditions, a power of 80% is achieved with 10 samples per condition.

### Molecular Analysis

- Sample preparation: Peripheral blood samples were collected from patients at T0 and T10, and just once from HC, and stored at – 20 °C in 3 ml PAXgene Blood RNA Tubes (PreAnalytiX Qiagen/BD, Hombrechtikon, Switzerland) until use. Total RNA was isolated using the PAXgene Blood RNA Kit (PreAnalytiX QIAGEN/BD, Hilden, Germany) at ITB CNR, Bari Unit. RNA concentration and purity were measured by Nanodrop ND-1000 (Thermo Scientific, Wilmington, DE, USA) and RNA 6000 Pico chip on Bioanalyzer 2100 (Agilent Technologies, Santa Clara, CA, USA), respectively. Samples with RNA Integrity Number (RIN) scores higher than 7 and with A260/A280 values in the 1.8–2.2 range were processed in downstream deep sequencing.
- High-throughput next-generation sequencing (HT-NGS): RNA samples were sequenced using an Illumina HiSeq2500 platform service (http://www.genomix4life.com/). The small RNAs/mRNA libraries were prepared using the TruSeq Small RNA Sample Preparation kit and the TruSeq Stranded mRNA Sample Preparation kit, respectively (Illumina, San Diego, CA, USA). The quality of both libraries was confirmed on a Bioanalyzer 2100 instrument (Agilent Technologies, Santa Clara, CA, USA). A multiplexed pool consisting of equimolar amounts of individual small-RNA-derived libraries was sequenced to generate 50-bp single-end reads, resulting in a final output of around 10 million reads per sample. The mRNAs libraries were pooled equimolar into a multiplex sequencing pool and sequenced to generate 2 × 100 bp paired end reads, leading to a final output of around 30 million reads per sample.

### Bioinformatic and Biostatistical Analysis

FASTQ files of mRNAs were mapped with STAR [PMID: 23104886] and RSEM [PMID: 21816040]. Read counts were computed with RSEM estimation and with MultiDEA [PMID: 28185579] uncertainty evaluation to annotate gene similarities that cause mapping uncertainty. SmallRNA (sRNA) FASTQ files were mapped with miRDeep2 [PMID: 21911355]. Differential expression analysis was performed with EBseq2, which automatically adapts expression evaluation for comparisons with and without replicates. The results were considered statistically significant with FDR ≤ 0.05 (PPDE > 0.95) and filtered using mean read count > 50 for longRNA (lnRNA) and > 20 for sRNA, and with absolute log2 Fold Change > 1 for lnRNA and > 0.585 for sRNA (corresponding to doubling and increase by one and a half times, respectively). The lists of inversely proportional differentially expressed genes and miRNAs were used for target evaluation in case and controls: underexpressed genes were evaluated as target of overexpressed miRNAs in the case, and overexpressed genes were evaluated as target of underexpressed miRNAs in the controls. miRNA-target gene interaction was evaluated using both experimentally validated miRNA–target interaction database (miRTarBase release 9.0: http://mirtarbase.mbc.nctu.edu.tw) and miRNA-target prediction algorithms (miRanda version 3.3a: http://www.microrna.org/microrna/home.do, accessed on June 2020; RNAhybrid version 2.1.2: https://bibiserv.cebitec.uni-bielefeld.de/rnahybrid; RNA22 version 2.0: https://cm.jefferson.edu/rna22; miRDB: https://mirdb.org/, accessed on September 2023; TargetScan release 8.0: http://www.targetscan.org/vert_80) applied on UTR data from Ensembl transcripts release-110 (Aken et al. 2016).

### Functional Analysis

Functional analysis was performed using the Database for Annotation, Visualization, and Integrated Discovery (DAVID, release 2023_04: https://david.ncifcrf.gov/home.jsp) (Dennis et al. 2003) that provides biological significance for the differentially expressed (DE) genes between SMA patients and healthy individuals. DAVID conducts an enrichment analysis using the Fisher Exact test and provides *p* values, along with other *p* values adjusted through various multiple correction methods (Benjamin, Bonferroni, and False Discovery rate, FDR). The tool utilizes various annotation databases, such as KEGG, Biocarta, Reactome, and the GO database.

Additionally, to improve the results, we conducted another enrichment test with G-profiler” (v0.2.2) (Kolberg et al. 2020). G-profiler2 also employs the Fisher exact test and provides *p*-adjusted values from various multiple correction tests. This tool encompasses an extensive array of databases, including KEGG, Reactome, GO, WikiPathways, miRTarBase, TRANSFAC, and CORUM.

### Network Analysis

The protein–protein interaction network of DE genes was constructed using String (v12.0: https://string-db.org/) (von Mering et al. 2003) and subsequent integration with the experimentally validated miRNA/mRNA interaction, as reported by miRTarBase. The network was built using *Cytoscape v3.10.1* (https://cytoscape.org/) (Shannon et al. 2003); *Cytohubba*, a Cytoscape plug-in, was utilized to identify hub genes in the molecular pathways resulted significant from our analysis (Chin et al. 2014). The main features considered from this tool were the *Degree* (= number of gene interactions), the Maximal Clique Centrality (*MCC*, it allows to measure the centrality of a node with respect to its membership in a subset of central nodes interconnected with each other), the Density of Maximum Neighborhood Component (*DMNC*: it quantifies the density of interactions of nodes close to a node of interest), the *Closeness centrality* (which measures the centrality of a node by taking into account how close it is to other nodes in the network), the Radiality (= how close a node is to the center of the network), the *Bottleneck* (it measures how important a node is in the network, and the impact it would have if it were removed from the network), the *Bet centrality* (it measures how much a node acts as an intermediary between other nodes), and the *Clustering coefficient*, which is the measure of how much a node tends to form clusters)(https://apps.cytoscape.org/apps/cytohubba).

## Results

**Fig. 1:**
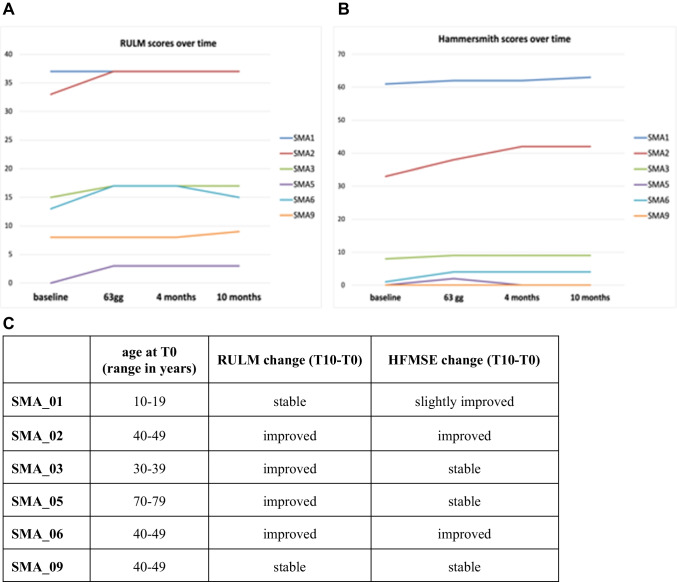
Summary of the neurological status during the 10 months observation for the 6 SMA patients under nusinersen who had useful NGS data in both T1 and T10 time points. In the top: graphical projection of the disability scores (**A** RULM score, **B** HFMSE score); **C** details of the disability changes [Responders were classified as patients who improved from baseline by at least 3 HFMSE points, 2 RULM points (Maggi et al. 2020). *SMA* spinal muscular atrophy, *RULM* revised upper limb module, *HFMSE* Hammersmith Functional Motor Scale Expanded]

The study population was composed of 10 patients (3 females and 7 males) affected by adult SMA (type 2–3); the individual demographic, genetic and clinical features of the recruited subjects are detailed in Table 1; overall, the mean age at the first symptom/s was 7.5 ± 5.2 years and the age at the study entry was 43.3 ± 13.5 years. Figure 1A–C summarizes the individual clinical changes registered during the study interval (T0–T10). None of the subjects exited the protocol of nusinersen administration during the study interval; one patient (SMA-5, a 73-year-old female) died for respiratory complication 8 months after the last study observation (T10). Further information about other biochemical markers measured during the study interval are available in Supplementary files.

**Table 1:** Baseline demographic, genetic, and clinical features of the SMA patients recruited for the study

Code	SMA type	*SMN1* variation	*SMN2* copies	Age of onset (yrs.)	Age range (yrs.)	Scoliosis	Spinal fusion	Weelchair	RULM	HFMS
SMA_01	3	HD	3	16	10–19	−	−	−	37	61
SMA_02	3	HD	4	10	40–49	+	−	−	33	33
SMA_03	2	HD	4	2	30–39	−	−	+	15	8
SMA_05	3	HD	3	10	70–79	+	−	+	0	0
SMA_06	2	CHV	3	3	40–49	+	−	+	13	1
SMA_07	3	HD	3	15	50–59	+	−	+	32	23
SMA_08	2	HD	3	2	30–39	+	−	+	19	7
SMA_09	2	HD	3	3	40–49	+	−	+	8	0
SMA_10	3	CHV	3	7	30–39	+	+	+	0	0
SMA_11	3	HD	3	7	40–49	−	−	−	37	44

Please note that actual age is expressed in range of years to avoid the patients’ identification and protect the individual right for privacy: (https://www.rpd.cnr.it/wp/?page_id=648)

*yrs*. years, *HD* homozygous deletion, *CHV* compound heterozygous variation, *RULM* revised upper limb module, *HFMSE* Hammersmith Functional Motor Scale Expanded

### SMN1/SMN2 Copies During Nusinersen

Since *SMN1* and *SMN2* have the entire coding sequence in common, apart from a single nucleotide change in exon 7 that causes the exon to be skipped, it is not easy to distinguish the origin of the NGS reads in control subjects (apart from those that match exon 7). On the other hand, in SMA subjects, in which *SMN1* is unproductive, all the reads associated by the mapper with a probabilistic calculation to *SMN1* actually come from *SMN2*. MultiDEA reported this ambiguity in the mapping, highlighting that almost 100% of the reads that mapped to one of these two genes also mapped identically to the other, while there was no ambiguity in these reads compared to other genes (no other genes showed significant portions in common with *SMN1* and *SMN2*).

**Fig. 2:**
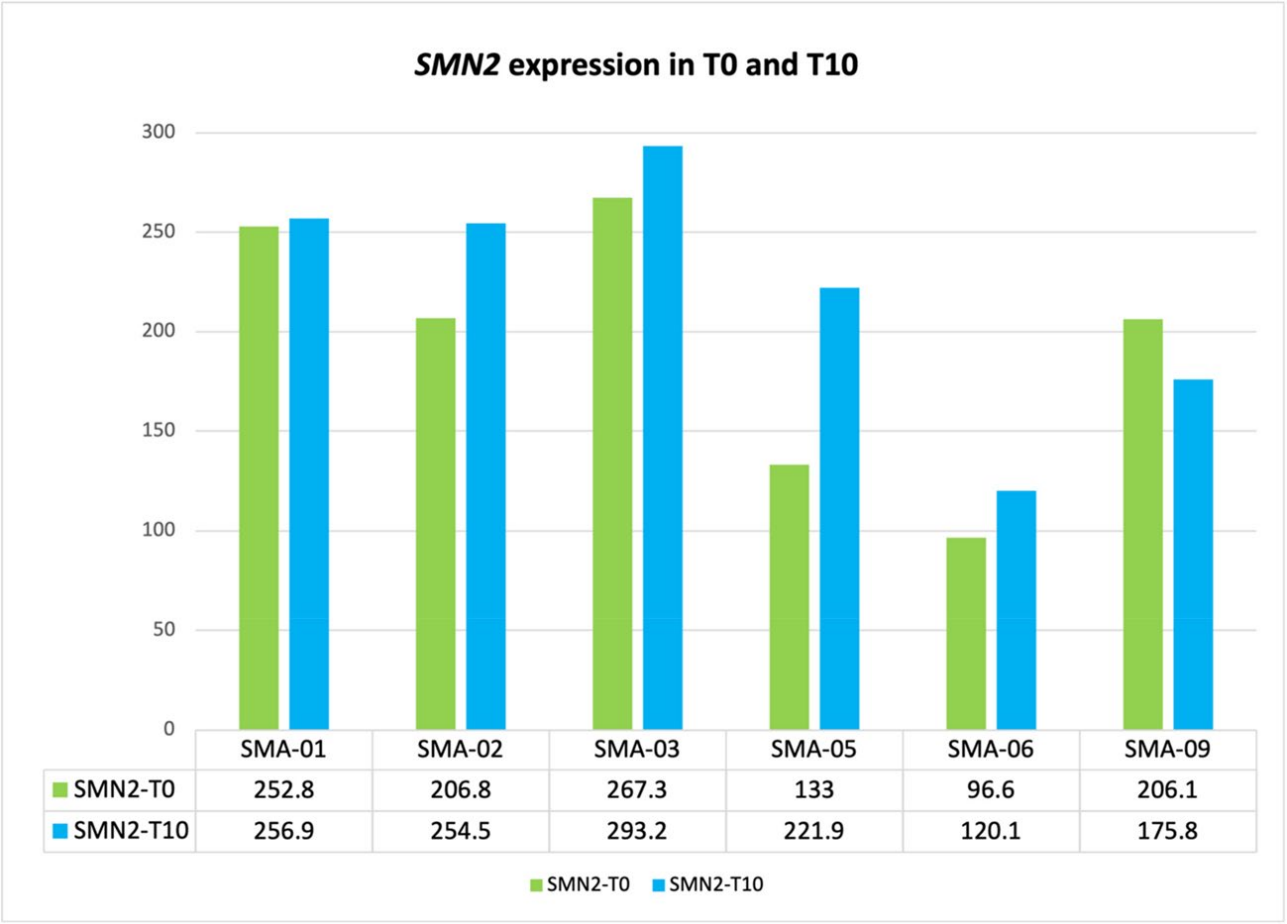
Changes in *SMN2* expression during nusinersen treatment. A significant increase (*p* value = 0.046) in *SMN2* expression is observed in all subjects except SMA_09 [Expression of *SMN2* at T0 and T1 estimated from RNA-seq data of SMA subjects (Y-axe = read counts normalized by EBSeq scaling factors]

Figure 2 shows the *SMN2* expression values obtained for SMA patients in T0 and T10. A statistically significant increase was observed (Wilcoxon one-tailed paired test, *p* value = 0.046), visible in all subjects except SMA_09, who is the only patient who showed a stable trend rather than a significant improvement in symptoms (Fig. 1C).

To evaluate the effect of the therapy also on the isoform level, we counted how many reads covered the junctions between exons6, exon7, and exon8, to look for an increase in the inclusion of the exon7 in patients (Table 2), but the counts obtained were rather low to make statistically significant conclusions. However, it is interesting to note that in patients with SMA the various isoforms are present even before therapy, which confirms the fact that the *SMN2* gene can produce functional proteins, although in fewer numbers than S*MN1*. In fact, although the low counts prevent the evaluation of their statistical significance, as expected no *SMN1* exon7 and no *SMN1* exon6-exon7 junction were found in the recruited SMA patients of this study. *SMN2* reads in HC can have a different proportion compared to *SMN1*, since *SMN2* copy number variations are present in throughout the population. We observed an increase of exon7 in almost all-time comparisons (FC > 1.5 highlighted in yellow—Table 3).

**Table 2:** Counting for the presence of exon7 in the reads produced by NGS, the following subsequences have been searched in the reads mapping *SMN1* and *SMN2* (in paired ends)

exon7 start from *SMN1*	GGTTT**C**AGACAAAAT
exon7 start from *SMN2*	GGTTT**T**AGACAAAAT
exon6-exon7 junction from *SMN1*	TATTATATG + GGTTT**C**AGA
exon6-exon7 junction from *SMN2*	TATTATATG + GGTTT**T**AGA
exon7-exon8 junction	AATTAAGGA + GAAATGCTG
exon6-exon7 and exon7-exon8 from SMN1*	TATATGGGTTT**C** & TAAGGAGAAATG
exon6-exon7 and exon7-exon8 from SMN2*	TATATGGGTTT**T** & TAAGGAGAAATG

**Table 3:**
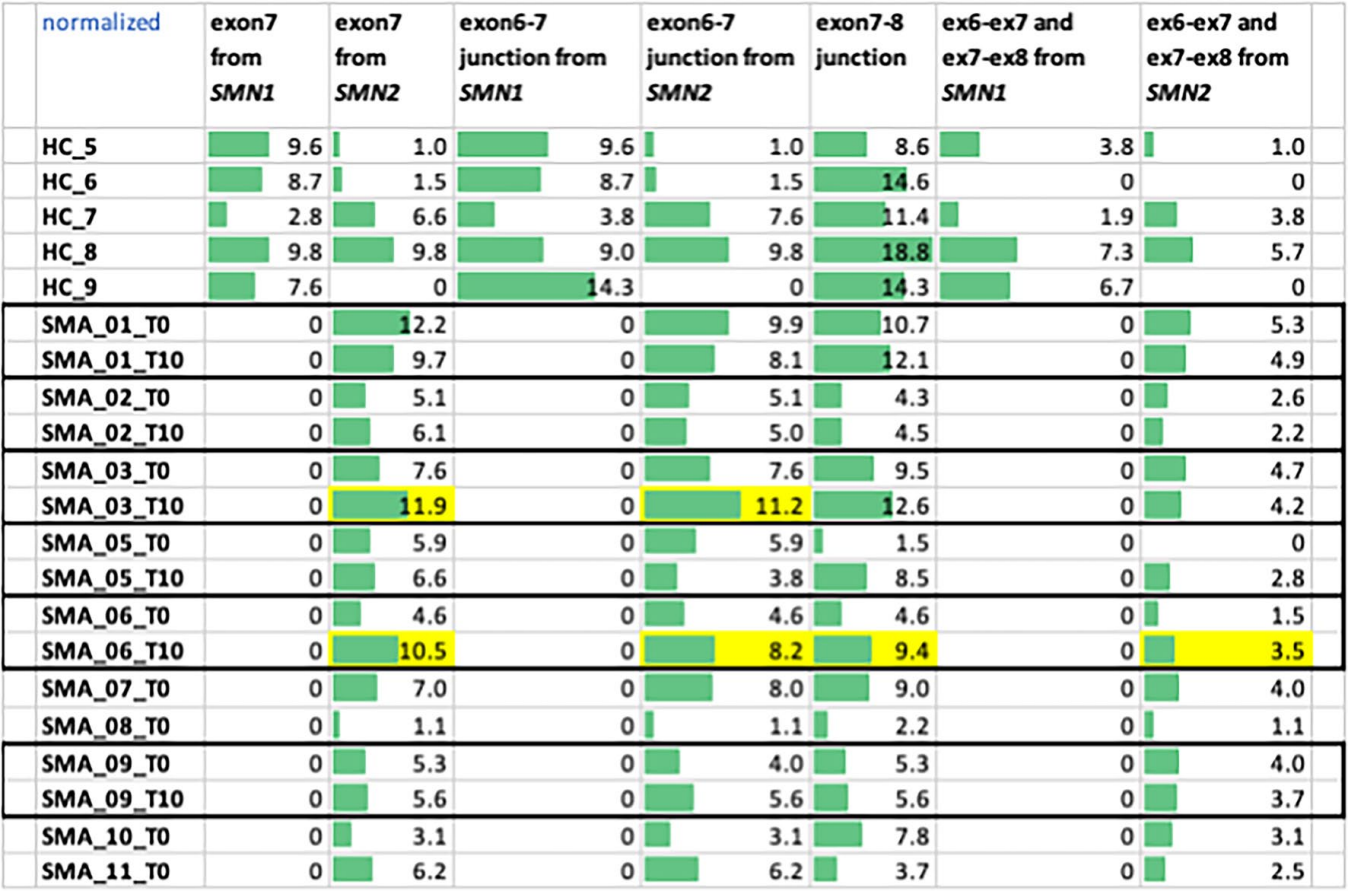
Data returned from HC and SMA subjects (T0 and T10, when available) and normalized with EBSeq scaling factors. *SMN2* reads in control subjects can have a different proportion compared to *SMN1*, since *SMN2* copy number variations are present in throughout the population (in yellow the exon7 increase when FC > 1.5)

### SMA (T0-T10) Versus HC

The quality check of HT-NGS returned admissible data only for 6 SMA patients with paired T0–T10 samples; in this cohort, the DE analysis of miRNA-seq did not find any significant common change in miRNA expression within all the paired T0–T10 samples, nor any correlation with the clinical scores (adj-*p* > 0.5). The analysis of mRNA-seq reads neither identified common significant DE genes/chromosomal regions between the two time points; however, each pair showed peculiar changes, ranging from a few (19 in SMA_01, 16 in SMA_02, 33 in SMA_03, 10 in SMA_06 and 6 in SMA_09) to 44 DE genes in SMA_05. Of note, in this last patient (female, age 73 years at T0, who died after T10), all the involved genes were downregulated within T10–T0 interval, like those related to the Integrin-mediated signaling pathway (individual mRNAs changes provided in Supplementary files).

The comparison between miRNA-seq reads of the whole group of the 10 recruited SMA patients at T0 (untreated) versus HC identified 39 significantly DE miRNAs, 19 downregulated and 20 upregulated. Of note, 10 of these miRNAs were no more significantly different at T10 (Table 4).

**Table 4:**
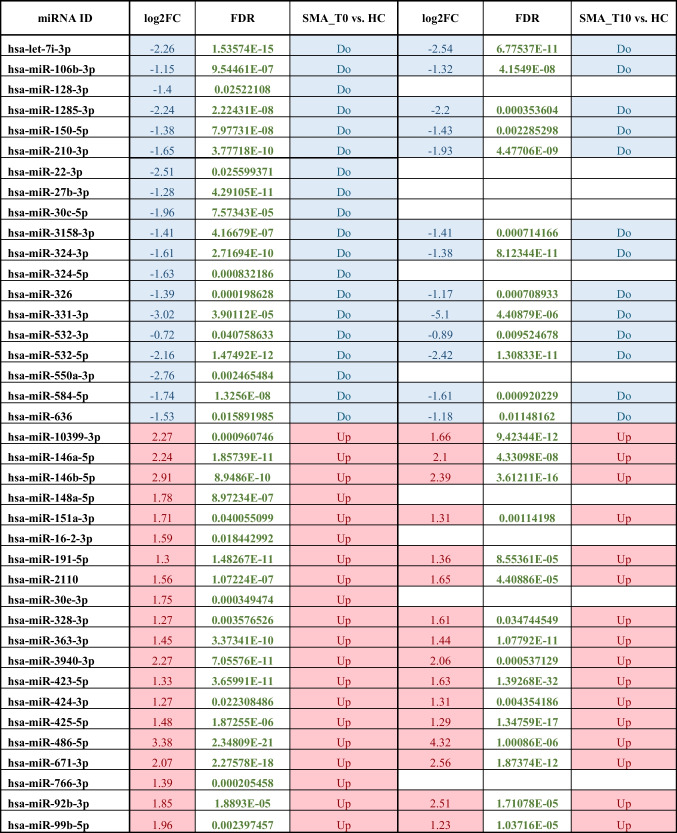
List of the significant DE miRNAs between SMA and HC. From the left, the first column listed the 39 miRNAs that resulted in the comparison between SMA patients (n. 10 at T0, naïve from specific treatments) and HC; the main attributes from the sRNA-seq analysis (log2-fold change, false discovery rate, FDR); the direction of the expression change (down/up). The last columns reported the respective data (log2FC, FDR) of the significant miRNAs expression in the SMA patients at T10 (n. 6 subjects) *versus* HC

The same comparison, performed on mRNA-seq data, revealed 147 DE genes (125 upregulated in SMA-T0 versus HC, the remaining 22 genes downregulated) (the complete list in Supplementary files); at T10, 38 of them did not differ anymore from HC (Table 5). Among the others, *TRADD* and *JUND* belonging to the IL-17 signaling pathway (by DAVID), and several genes encoding for zinc fingers proteins like *ZNF524, ZNF467, ZNF628*, and *ZNF579*.

**Table 5:** Gene expression changes during nusinersen. List of the 38 genes resulted significantly DE between SMA_T0 (naïve from specific treatments) and HC, but not after 10 months treatment (T10). The table summarizes the main attributes from the sRNA-seq analysis (log2-fold change, false discovery rate, FDR); the direction of the expression change (down/up). For the complete list of RNAs compared to HC at both time points, see Supplementary Files

Ensembl ID		log2FC	FDR	SMA T0 vs HC
ENSG00000164649	***CDCA7L***	− 1.09	**0.016198224**	Do
ENSG00000179978	***NAIPP2***	− 1.6	**0.001674627**	Do
ENSG00000187922	***LCN10***	− 1.19	**0.003737614**	Do
ENSG00000251201	***TMED7-TICAM2***	− 1.29	**0.000242708**	Do
ENSG00000280734	***LINC01232***	− 1.03	**0.045443269**	Do
ENSG00000007520	***TSR3***	1.02	**2.93E-10**	Up
ENSG00000025708	***TYMP***	1.22	**0.001129937**	Up
ENSG00000070423	***RNF126***	1	**2.83E-12**	Up
ENSG00000102871	***TRADD***	1.04	**4.89E-10**	Up
ENSG00000105327	***BBC3***	1.22	**3.45E-26**	Up
ENSG00000105404	***RABAC1***	1.16	**4.04E-09**	Up
ENSG00000105655	***ISYNA1***	1.02	**7.32E-06**	Up
ENSG00000108479	***GALK1***	1.1	**9.16E-05**	Up
ENSG00000110628	***SLC22A18***	1.1	**0.00013788**	Up
ENSG00000123144	***TRIR***	1.05	**9.71E-30**	Up
ENSG00000124074	***ENKD1***	1.01	**0.015046952**	Up
ENSG00000125910	***S1PR4***	1.12	**1.49E-12**	Up
ENSG00000128228	***SDF2L1***	1.18	**7.35E-09**	Up
ENSG00000129757	***CDKN1C***	1.74	**0.032920657**	Up
ENSG00000129968	***ABHD17A***	1.13	**3.93E-05**	Up
ENSG00000130522	***JUND***	1.09	**6.44E-14**	Up
ENSG00000135722	***FBXL8***	1.47	**0.010988137**	Up
ENSG00000150045	***KLRF1***	1.11	**0.001649658**	Up
ENSG00000158106	***RHPN1***	1.01	**5.09E-06**	Up
ENSG00000160813	***PPP1R35***	1.02	**1.10E-12**	Up
ENSG00000170604	***IRF2BP1***	1.08	**1.44E-10**	Up
ENSG00000170638	***TRABD***	1.07	**1.59E-17**	Up
ENSG00000171443	***ZNF524***	1.13	**1.16E-06**	Up
ENSG00000176973	***FAM89B***	1.01	**1.15E-07**	Up
ENSG00000181444	***ZNF467***	1.59	**0.000122306**	Up
ENSG00000182087	***TMEM259***	1.03	**1.40E-21**	Up
ENSG00000182154	***MRPL41***	1.11	**1.39E-06**	Up
ENSG00000182809	***CRIP2***	1.3	**0.044632487**	Up
ENSG00000197483	***ZNF628***	1.91	**2.30E-17**	Up
ENSG00000197530	***MIB2***	1.23	**7.46E-05**	Up
ENSG00000204839	***MROH6***	1.11	**0.008771969**	Up
ENSG00000213563	***C8orf82***	1.08	**1.48E-06**	Up
ENSG00000218891	***ZNF579***	1.94	**5.01E-16**	Up
ENSG00000240972	***MIF***	1.09	**1.54E-07**	Up
ENSG00000257704	***INAFM1***	1.22	**5.56E-10**	Up
ENSG00000265666	***RARA-AS1***	1.15	**0.008065409**	Up

### Validated and Predicted miRNA-Target Interaction

The analysis evaluating the interactions between up-regulated miRNAs and downregulated genes showed that *SMN1*, *HLA-DRB1*, *KLF2*, and *IFNLR1* were validated targets of miR-146a-5p, miR-148a-5p, miR-532-3p, and miR-151a-3p, respectively. Table 6 also shows the results of the down-regulated miRNAs and their validated upregulated genes; among the others, *SMN1* was also identified as possible gene target of miR-766-3p, whereas *IFNRL1* was found as predicted target of miR-328-3p and miR-3940-3p.

**Table 6:** Validated or predicted genes targeted from the significant miRNAs. In the first columns (from the left), the Ensembl ID and the common gene symbol; the third column listed the miRNAs that targeted the gene, followed by the type of interaction (either is only the prediction from our bioinformatic analysis and/or the published experimental validation); the direction of both the expressions, and the citation of the genes. Note that the table represents both the results of the primary analysis (in green: the miRNA was overexpressed in SMA versus HC, while its target gene was down regulated), as those of the secondary analysis (*in yellow:* the miRNA’ expression was significantly lower in SMA while its target gene was over-expressed)

Target gene (Entrez ID)	Gene symbol	miRNA	Type of interaction	miRNA expression*	Gene expression*	Citations of the gene
ENSG00000272410	*–*	hsa-miR-10399-3p	*predicted*	*up*	*down*	
	*–*	hsa-miR-2110	*predicted*	*up*	*down*	
ENSG00000285304	*–*	hsa-miR-423-5p	*predicted*	*up*	*down*	
	*–*	hsa-miR-3940-3p	*predicted*	*up*	*down*	
	*–*	hsa-miR-671-3p	*predicted*	*up*	*down*	
ENSG00000198898	*CAPZA2*	hsa-miR-150-5p	validated	down	Up	
ENSG00000164649	*CDCA7L*	hsa-miR-92b-3p	*predicted*	*up*	*down*	*MDD*
ENSG00000129932	*DOHH*	hsa-miR-331-3p	validated	down	Up	Neurodevelopment disorders
ENSG00000196126	*HLA-DRB1*	hsa-miR-148a-5p	validated	Up	down	AID, AD, PD
ENSG00000185436	*IFNLR1*	hsa-miR-151a-3p	validated	Up	down	autophagy
	*IFNLR1*	hsa-miR-328-3p	*predicted*	*up*	*down*	
	*IFNLR1*	hsa-miR-3940-3p	*predicted*	*up*	*down*	
ENSG00000274049	*INO80B-WBP1*	hsa-miR-328-3p	*predicted*	*up*	*down*	
	*INO80B-WBP1*	hsa-miR-423-5p	*predicted*	*up*	*down*	
ENSG00000261796	*ISY1-RAB43*	hsa-miR-328-3p	*predicted*	*up*	*down*	
	*ISY1-RAB43*	hsa-miR-423-5p	*predicted*	*up*	*down*	
	*ISY1-RAB43*	hsa-miR-92b-3p	*predicted*	*up*	*down*	
	*ISY1-RAB43*	hsa-miR-99b-5p	*predicted*	*up*	*down*	
ENSG00000127528	*KLF2*	hsa-miR-532-3p	validated	down	Up	AD, DMD
ENSG00000187922	*LCN10*	hsa-miR-2110	*predicted*	*up*	*down*	
	*LCN10*	hsa-miR-423-5p	*predicted*	*up*	*do*	
	*LCN10*	hsa-miR-766-3p	*predicted*	*up*	*do*	
ENSG00000128011	*LRFN1*	hsa-miR-324-3p	validated	down	Up	
ENSG00000130881	*LRP3*	hsa-miR-331-3p	validated	down	Up	AD, apoptosis
ENSG00000130881	*MGAT2*	hsa-miR-30c-5p	validated	down	Up	Brain developmental disorders
ENSG00000173272	*MZT2A*	hsa-miR-324-5p	validated	down	Up	
ENSG00000103024	*NME3*	hsa-miR-324-5p	validated	down	Up	Mitochondrial disorders, AD
ENSG00000105404	*RABAC1*	hsa-miR-150-5p	validated	down	Up	
ENSG00000128228	*SDF2L1*	hsa-miR-30c-5p	validated	down	Up	AD
ENSG00000172062	*SMN1*	hsa-miR-146a-5p	validated	Up	down	**SMA**
	*SMN1*	hsa-miR-766-3p	*predicted*	*up*	*down*	
ENSG00000140406	*TLNRD1*	hsa-miR-27b-3p	validated	down	Up	
	*TLNRD1*	hsa-miR-128-3p	validated	down	Up	
ENSG00000180233	*ZNF703*	hsa-miR-550a-3p	validated	down	Up	
ENSG00000183780	*ZNRF2*	hsa-miR-326	validated	down	Up	

*MDD* major depressive disorder, *ND* neurodegeneration, *AID* autoimmune diseases, *AD* Alzheimer’s disease, *PD* Parkinson’s disease, *DMD* Duchenne muscular atrophy, *SMA* spinal muscular atrophy. *Our data

**Fig. 3:**
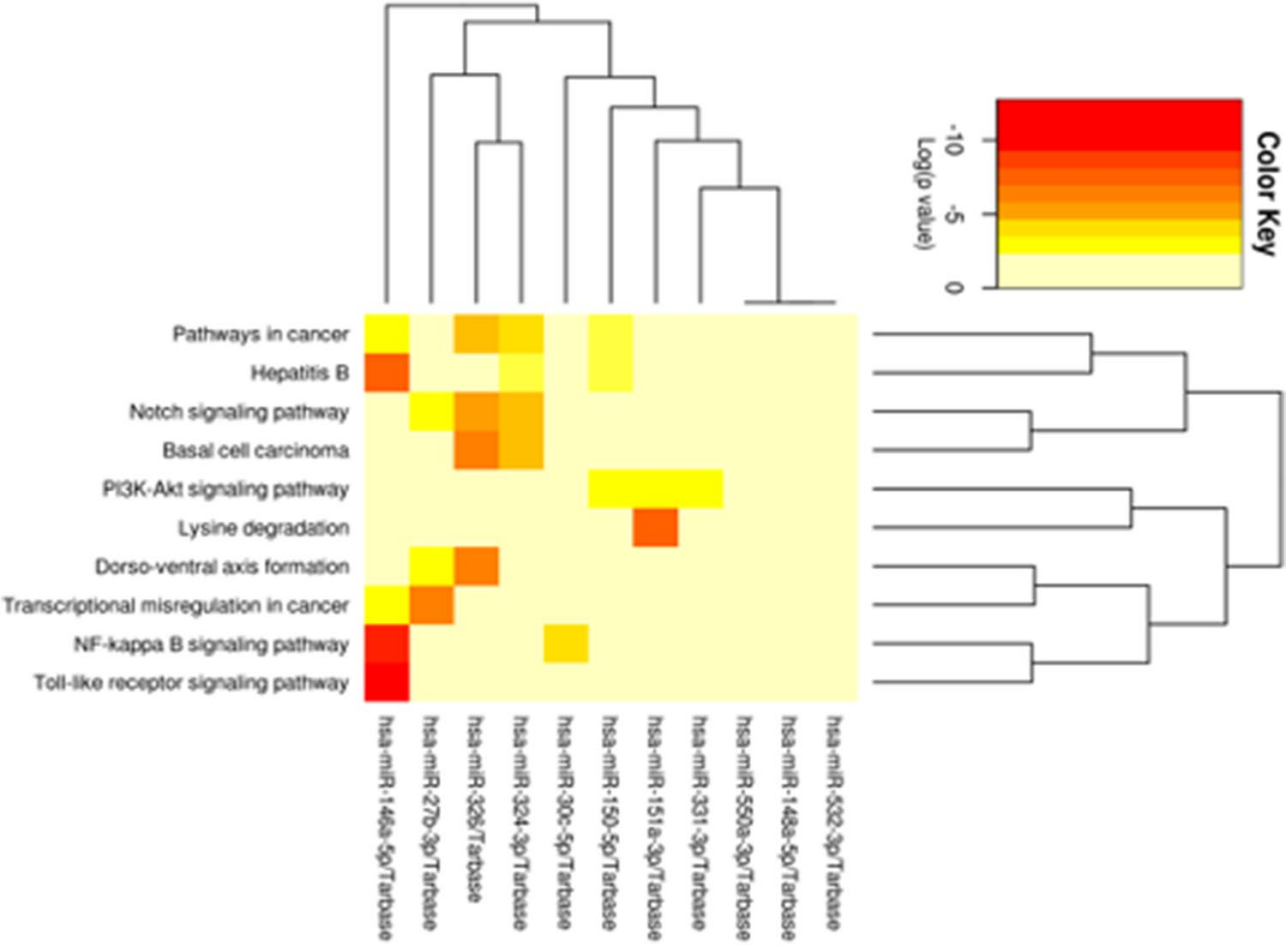
Heatmap in SMA-T0 vs HC representing DE miRNAs that targeted DE genes (validated results by references) (Vlachos et al. 2012)

Although some of these interactions have been already validated and evoked interesting functional pathways like the NOTCH and NF-kappa signaling, and the Toll-like receptor signaling (see Fig. 3), those remaining seek for experimental confirmations.

### Functional Analysis and Molecular Network

**Fig. 4:**
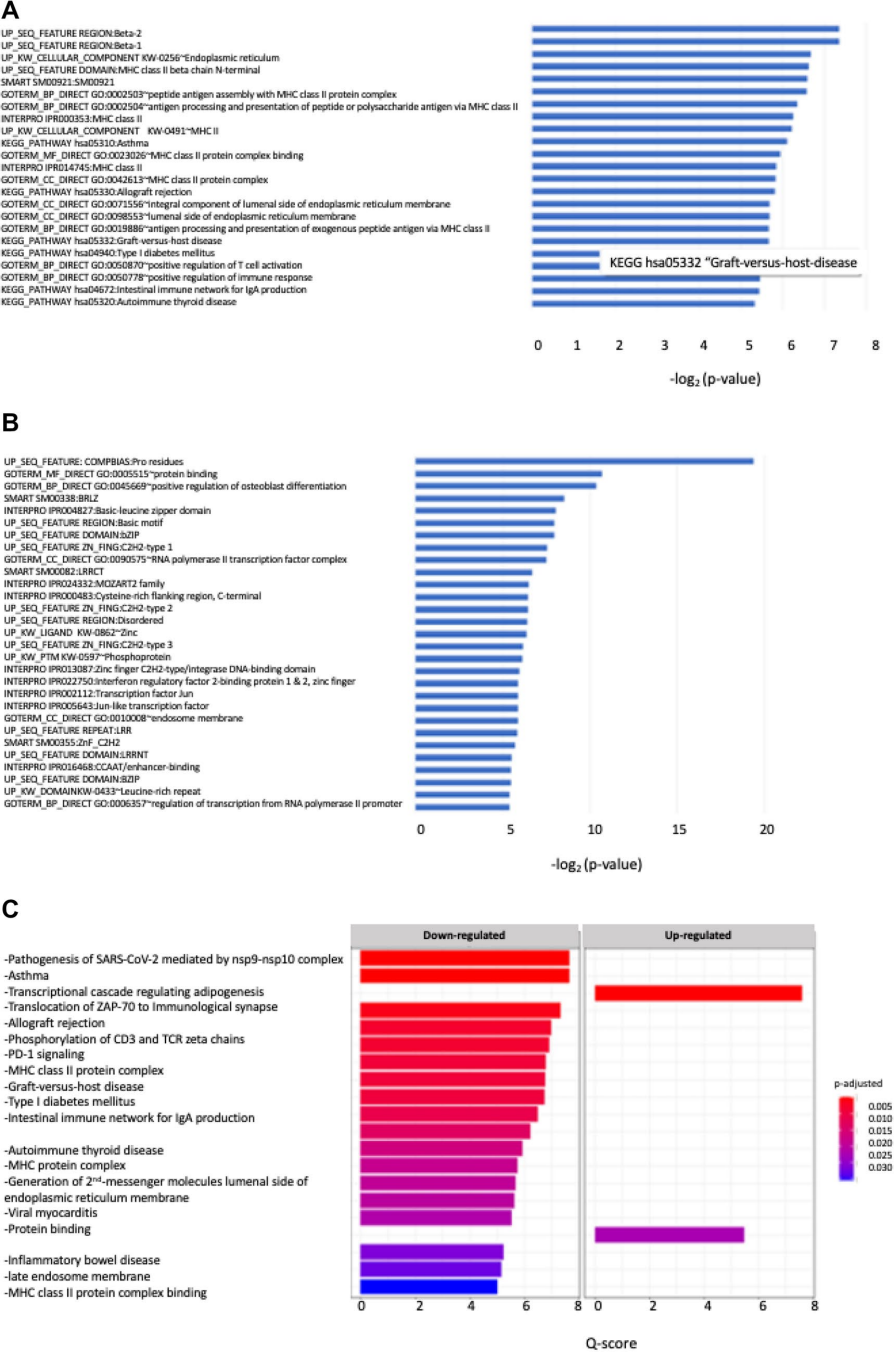
Significant pathways (*p* < 0.025) involving the downregulated genes in HC with the corresponding -log2(*p* value). **A**, **B** Functional analysis was performed using the database for annotation, visualization, and integrated discovery (DAVID) to provide biological significance for the differentially expressed (DE) genes between SMA patients and healthy individuals. **C** Representation of enriched terms both up and downregulated resulting from Gprofiler2 (see “Materials and Methods” section for details) with their corresponding qscore (log2 (*p*-adjust)

By looking at the down/upregulated genes in SMA-T0 patients compared to HC (DAVID), data showed that no significant enriched functional annotations survived the correction for multiple tests (Fig. 4A and B). However, the search for functional clusters identified several terms involved in the downregulation of the immune system during SMA (i.e., GO:0002505, GO:0002504, GO:0023026), as well as the upregulation of osteoblast differentiation (GO:0045669), and of transcription factors (TFs)*.* It is worthy to note that these results were confirmed and found to be significant by the G-profiler2 analysis (Fig. 4C).

**Fig. 5:**
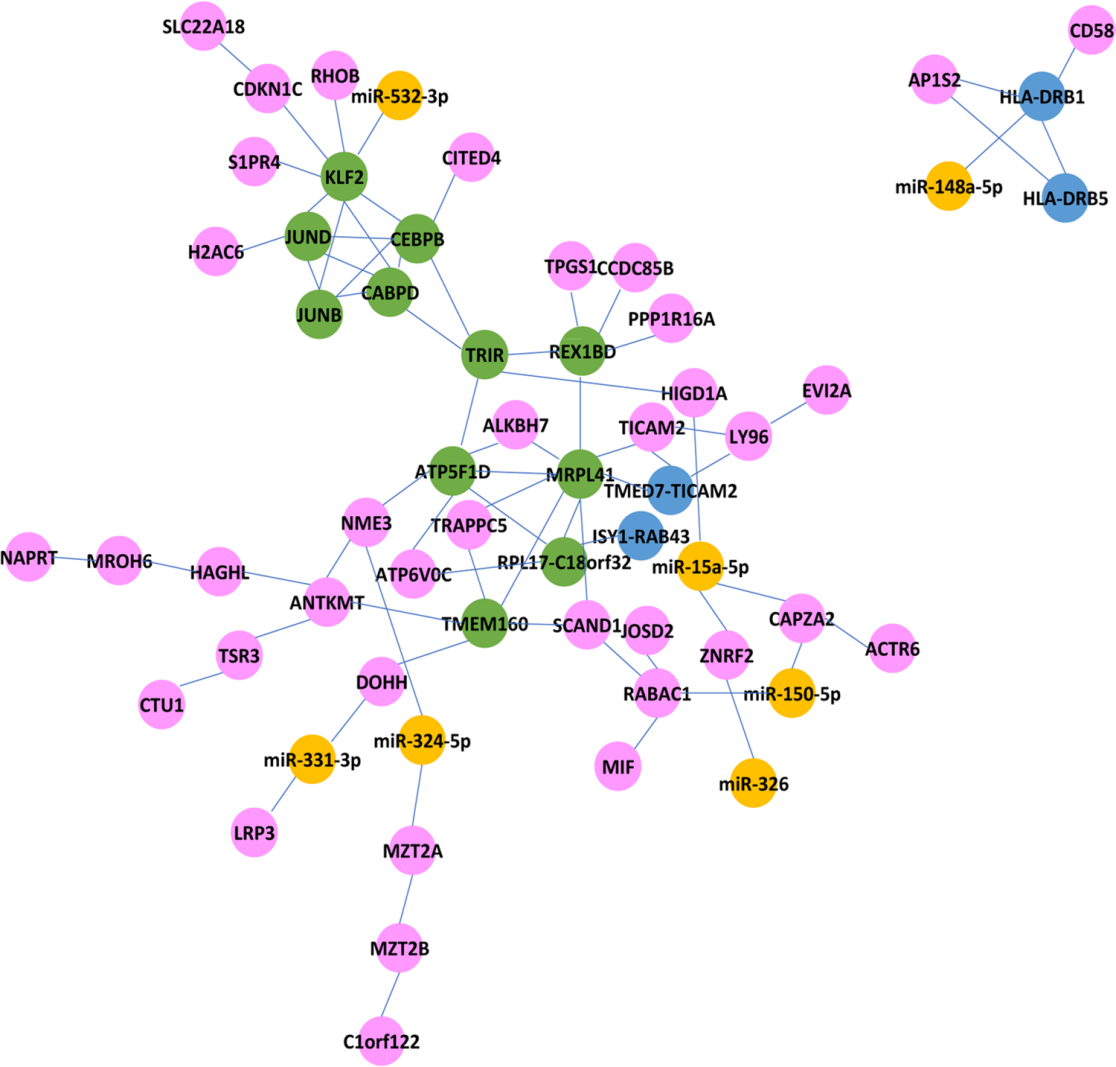
Molecular network generated by the combination of genes up/downregulated in SMA-T0 versus HCs, significant transcription factors (TFs), and validated DE miRNAs [Genes downregulated (*blue*), genes upregulated (*pink*), and the top 11 hub genes (*green*—details in the Table below). In *yellow*, those miRNAs that targeted the significant DE genes (validated) (by Cytohubba—see “Material and Methods” section for further explanation)]


Figure 5 shows the molecular network that involves the significant miRNAs, their targeted genes and the hub-genes. Among the interesting network, hsa-miR-148a-5p and *HLA-DRB1 related to HLA-DRB5*, *AP1S2* and *CD58*, and hsa-miR-532-5p that seem related to the net composed by *KLF2*, *CEBPD*, *CEBPB*, *JUND*, and *JUNB* (respective log2FC: 1.66, 3.07, 1.89, 1.09 and 1.36; FDR < 0.00001). Of interest, the search for the top 10 hub-genes (by *Cytohubba*), which likely play a crucial role in the molecular network involved in SMA, identified intriguing molecular interconnections (see Table enclosed to Fig. 5); for instance, *MRLP41*, *TRIR*, and *ATP5F1D* displayed a high betweenness centrality and bottleneck values, suggesting their key roles in the interconnection with other nodes. Interestingly, *KLF2*, *TMEM160*, and *CEBPD* were significantly upregulated among all the analyzed SMA patients (Supplementary files).

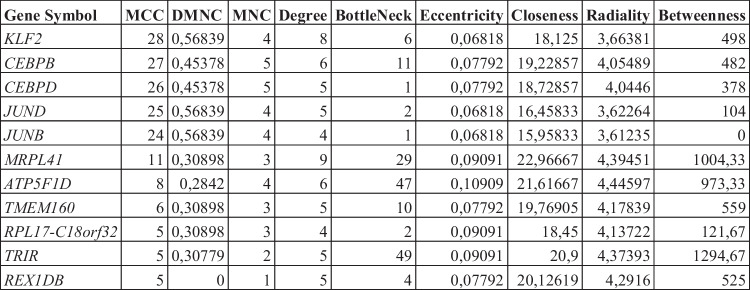


## Discussion

In this 10-month longitudinal study, the HT-NGS transcriptomic analysis performed on adult SMA patients showed several features possibly related to the nusinersen administration that, in our view, deserve some attention.

The first note concerns the estimation of *SMN2* copies analyzed early in the T0-T10 interval. Data showed that in patients with some clinical improvement in one or both the disability scales (SMA_01, SMA_02, SMA_03 and SMA_06), the *SMN1*/*2* ratio slightly increased over the time. On the other hand, in the stable SMA_09, the ratio decreased, suggesting that the treatment was not as beneficial as in the other patients, while in SMA_05 (whose prognosis was rapidly fatal) we may hypothesize that the increase in *SMN2* expression was not sufficient to counteract the progress of the pathology. Some influencing factors should be considered for explaining the latter finding, above all the age at nusinersen administration (73 years old in SMA_05) that is of course closely connected to the disease duration; and of course, 10 months is a short interval for conclusive statement on treatment responsiveness. Nevertheless, as far as we know, this is the first study reporting the *SMN2*/*SMN1* expression changes during nusinersen in adult SMA. If confirmed in a larger cohort, this data may lead to recommend the estimation of *SMN2*/*SMN1* expression as early indicator for nusinersen efficacy in adult SMA, which may be a valuable support for the clinical practice, considering the administration route of this therapy.

It is also worth mentioning that 38 genes that resulted DE in SMA patients at T0 compared to HC were no longer different at T10. As an example, the upregulated *TRADD* and *JUND* were “normalized” at T10, so we may speculate that nusinersen was able to impact, e.g., the IL-17 signaling pathway in which both the genes are involved, which is in line with the potential of this therapy to mitigate the neuroinflammatory features of SMA (Nuzzo et al. 2023a). Another interesting tip comes from the involvement of genes encoding for zinc-finger proteins that are known potential modifiers of SMA (Kannan et al. 2020). Although these data need functional confirmation in the long-term treatment and larger samples, we believe that they add some novel insights in the molecular evaluation of the effect of nusinersen, as well as they suggest that peculiar molecular pathway may drive the lack of its efficacy, i.e., the upregulation of the Integrin-mediated signaling pathway in SMA_05, which may be a negative prognostic signature (Delers et al. 2022).

As the second goal of the investigation, we found several intriguing results that seem to characterize the molecular profile of adult SMA patients compared to age-matched HCs. Among the significant miRNAs, we confirmed the dysregulation of miR-146a(-5p) (Sison et al. 2017), miR-324-5p (Abiusi et al. 2021), and miR-423(-5p) (Zaharieva et al. 2022) in our SMA subjects. Of interest, miR-146a-5p targeted *SMN1*, the miRNAs resulted significantly overexpressed while the gene was downregulated. To our view, this finding is in line with a recent report discussing the experimental role of astrocyte-produced miR-146a in the motor neuron loss that characterized SMA. In particular, the study revealed a significant increase of miR-146a in SMNΔ7 mouse spinal cord; furthermore, when iPSC-derived motor neurons were treated with synthesized miR-146a, it seemed to induce significant motor loss, whereas this process was blocked by miR-146a inhibitor (Sison et al. 2017). The authors hypothesized that the mechanism upregulating mir-146a may be trough GATA transcription factors, mainly *GATA6*, which was found highly expressed in SMA mouse and human samples and correlated with the disease severity, or via *NOTCH2* impact (Yang et al. 2016). It has been also demonstrated that NFkB interact synergistically with GATA6, and this interplay leads to activation of miR-146a (Boopathi et al. 2013), which is consistent with the upregulation of NFkB observed in the SMA iPSC-derived astrocytes (Sison et al. 2017).

Neither of these regulations clearly explain the astrocyte malfunctions observed in SMA pathology, nor they showed a direct effect on *SMN1*. Although without experimental validation, we believe that our extensive analysis added few tips in this view; in fact, the heatmap of the most significant pathways involved by the 13 DE miRNAs (enclosing miR-146a-5p), which targeted validated DE genes in SMA *versus* HCs (Table 5), confirmed the involvement of both Notch and NFkB signaling (Fig. 3). Most important, the bioinformatics analysis showed that miR-146a significantly targeted *SMN1.* Functional validations need to follow to verify this pathogenic mechanism.

On the other hand, in our analysis, some genes were targeted by several miRNAs, and their roles emerged as particularly interesting in the scenario of NDs like SMA. Among the others, *IFNLR1* was significantly downregulated in SMA as they were targeted by overexpressed miR-151a-3p, miR-125a-5p, miR-328-3p, and miR-3940-3p. Although only the first 2 pairs were validated results by literature—the remaining being predicted by our bioinformatics analysis—this data suggest that *IFNLR1* may in fact be relevant in SMA pathogenesis. Of note, literature data reported that IFN-λ receptor (*IFNLR1*) deficiency was associated to significantly impact of the immune cells’ activation and to the skin and kidneys damage without effects on autoantibody production (Goel et al. 2020), suggesting that an “immune interference” should be considered in SMA. Several recent evidence pointed in fact towards an immune dysregulation in SMA, as SMN seem to have a central role for the healthy development of the lymphoid system (Deguise et al. 2017), as well as specific drugs like nusinersen have been reported to improve some features more likely related to neuroinflammation (Bonanno et al. 2022; Nuzzo et al. 2023b).

Indeed, the functional bioinformatic analysis performed in our study pointed to a significant downregulation of the immune system during SMA (Fig. 4A–C) represented by enriched terms concerning, e.g., MHC class II protein complex and binding, auto/immune diseases (thyroid, type-1 diabetes, graft-versus host), intestinal immune network for IgA production. It is also noteworthy that one of the molecular networks that resulted from the functional analysis (Fig. 5) enclose *HLA-DRB1* and *HLA-DRB5* that have been confirmed implicated in MS and other autoimmune diseases (Irizar et al. 2012; Agliardi et al. 2023) as well as in AD and other NDs (Hampel et al. 2020). In depth analysis still needs to be done also in this direction, taken for overturned the past assumption that SMA is just a motor neuron disease (Yeo and Darras 2020).

We are aware that the study suffers for some limitations, as the small sample size and the heterogeneity of the recruited patients, whose age at first nusinersen administration ranged from 19 to 73 years old, which leads to a very wide changes in the disease duration and clinical disabilities, with subsequent differences of the individual transcriptomic profile (Schaum et al. 2020; Rutledge et al. 2022). Unfortunately, considering the way of administration and the evidence that nusinersen is rapidly effective mainly in infant SMA (Finkel et al. 2017; De Vivo et al. 2019), only few adult patients agreed to be treated, so we were not able to minimize these variables. On the other hand, although a recent study on children and adolescent SMA patients did find significant DE miRNAs after the first 6 month of nusinersen therapy, which is consistent with the dramatic improvement of clinical disability (Zaharieva et al. 2022), it is reasonable to believe that in our adult cohort a 10-month interval might be a quite short time for uncovering significant transcriptomic changes as for clinical improvement, if any. A longer observation with clinical scales for adult subjects that will be able to provide more significant information (e.g., about changes in daily activities) should help in this purpose (Maggi et al. 2020); however, we must disclose that during this observation another specific drug (Risdiplam) became available as oral therapy authorized in Italy also for the adult SMA. Since most of our recruited subjects expressed their will to shift the therapy, given the complexity of the nusinersen administration, it would be rather difficult to extend this transcriptomic analysis to further time points.

Nevertheless, we believe that combined miRNAs/mRNAs expression analysis may be crucial for a more comprehensive approach also in rare genetic diseases like SMA, in which the causative mutation is known but so far, the whole pathogenic mechanism still needs some clarification.

Looking at the whole picture, an interesting network was in fact depicted from our resulting data (Fig. 5) in which some hub-genes seem to exert crucial roles, as they had central position in the connection between the nodes. As an example, we found that *MRLP41*, encoding for the protein BMRP with pro-apoptotic activity (by binding Bcl-2) (Malladi et al. 2011), was related to 9 others (*TICAM2*, *ATP5F1D*, *TRAPPC5*, *SCAND1*, *ALKBH7*, *TMEM160*, *TMED7-TICAM2*, *REX1BD*, *RPL17-C18orf32*) with a high bottleneck coefficient suggesting a fundamental role of this gene in the SMA molecular network.

Other information derives from the net composed by 5 TFs (*KLF2*, *CEBPB*, *CEBPD*, *JUND*, and *JUNB*), all upregulated and closely implicated in NDs like AD, ALS, SCA, and PD (Evert et al. 2006; Wu et al. 2013; Doxakis 2020; Sun et al. 2022; He et al. 2023). Since depletion of *KLF2* (the encoding gene being one of the top genes with highest MCC score measuring its centrality in the net) was reported to cause enhanced apoptosis NGF-mediated (Dutta et al. 2011), while overexpression of the two TFs *CEBPB* and *CEBPD* significantly characterized ALS (Sun et al. 2022), and *JUND* is one of the “allegedly restored” gene by nusinersen in our study, it is reasonable to hypothesize that the interaction between them—possibly mediated by miRNAs like miR-532-3p—could be critically involved also in SMA.

Investigation on larger cohorts together with functional validations should be performed to possibly confirm the role of these novel molecular hotspots toward addressing more therapeutic efforts.

## Supplementary Information

Below is the link to the electronic supplementary material.

Supplementary file1 (DOCX 121 KB)

## Data Availability

No datasets were generated or analysed during the current study.
