# Supplementary material for: An early Transcriptomic Investigation in Adult Patients with Spinal Muscular Atrophy Under Treatment with Nusinersen
Source: J Mol Neurosci. 2024 Sep 26;74(4):89. doi: 10.1007/s12031-024-02251-1 (PMC11427494; doi:10.1007/s12031-024-02251-1)
Supplement: Supplementary file 1 — Supplementary file1 (DOCX 121 KB) [file 12031_2024_2251_MOESM1_ESM.docx]

**Supplementary Table 1:** CSF measurement of neurodegeneration biomarkers in the study SMA population by solid‐phase enzyme immunoassay (INNOTEST; Fujirebio, Ghent, Belgium). As indicated in the second half of the table, the paired analysis between T0 and T10 did not find significant changes.

|  | **Median** | **IQR** |
| --- | --- | --- |
| **Amyloid β1-40 (T0)** | 3800 | (3600-4900) |
| **Amyloid β1-40 (T10)** | 5355 | (4500-5510) |
| **Amyloid β1-42 (T0)** | 398 | (305-613) |
| **Amyloid β1-42 (T10)** | 438 | 357-588) |
| **Amyloid β1-42/ Aβ1-40 (T0)** | 0.08 | (0.08-0.13) |
| **Amyloid β1-42/ β1-40 (T10)** | 0.11 | (0.08-0.12) |
| **Neurofilament light chain (T0)** | 436 | (406-577) |
| **Neurofilament light chain (T10)** | 318 | (288-414) |
| **Tau (T0)** | 109 | (90-172) |
| **Tau (T10)** | 98 | (82-151) |
| **pTau (T0)** | 16 | (16-37) |
| **pTau (T10)** | 16 | (16-16) |

| Wilcoxon paired test between T0-T10: | | | | | | | | | |
| --- | --- | --- | --- | --- | --- | --- | --- | --- | --- |
|  | |  | |  | |  | | **p** | |
| Amyloid β1-40 |  | - |  |  |  |  |  | 0.7482 |  |
| Amyloid β1-42 |  | - |  |  |  |  |  | 0.3383 |  |
| Amyloid β1-42/ Aβ1-40 |  | - |  |  |  |  |  | 0.65 |  |
| Neurofilament light chain |  | - |  |  |  |  |  | 0.1541 |  |
| Tau |  | - |  |  |  |  |  | 0.2650 |  |
| pTau |  | - |  |  |  |  |  | 0.5217 |  |
|  | | | | | | | | | |

***Abbreviation:***

Aβ= amyloid‐β peptide; Aβ40= amyloid‐β40 peptide; Aβ42= amyloid‐β42 peptide; pTau= Phospho-Tau

***Biochemical methods in:*** Introna A, Milella G, D'Errico E, Fraddosio A, Scaglione G, Ucci M, Ruggieri M, Simone IL. Is cerebrospinal fluid amyloid-β42 a promising biomarker of response to nusinersen in adult spinal muscular atrophy patients? Muscle Nerve. 2021 Jun;63(6):905-909. doi: 10.1002/mus.27212.

***Supplementary-Table 2:*** completed list of RNAs (219 genes and 36 novel transcripts that resulted from the comparisons between SMA patients *versus* HC (both the time points).

|  |  | **log2FC** | **FDR** | **SMA_T0 vs HC** | **log2FC** | **FDR** | **SMA_T10 vs HC** |
| --- | --- | --- | --- | --- | --- | --- | --- |
| ENSG00000000419 | ***DPM1*** | 1.09 | **4.87804E-09** | Up |  |  |  |
| ENSG00000006015 | ***REX1BD*** | 1.45 | **1.25172E-23** | Up | 1.24 | **3.18041E-40** | Up |
| ENSG00000007520 | ***TSR3*** | 1.02 | **2.934E-10** | Up |  |  |  |
| ENSG00000025708 | ***TYMP*** | 1.22 | **0.001129937** | Up |  |  |  |
| ENSG00000064225 | ***ST3GAL6*** |  |  |  | 1.02 | **3.83706E-07** | Up |
| ENSG00000065243 | ***PKN2*** |  |  |  | 1.15 | **1.1885E-09** | Up |
| ENSG00000067167 | ***TRAM1*** |  |  |  | 1.03 | **1.93693E-12** | Up |
| ENSG00000068885 | ***IFT80*** |  |  |  | 1.00 | **0.001554773** | Up |
| ENSG00000070423 | ***RNF126*** | 1.00 | **2.83459E-12** | Up |  |  |  |
| ENSG00000073150 | ***PANX2*** | 1.49 | **8.53629E-05** | Up | 1.10 | **0.012991063** | Up |
| ENSG00000075089 | ***ACTR6*** |  |  |  | 1.37 | **5.52736E-11** | Up |
| ENSG00000086065 | ***CHMP5*** |  |  |  | 1.08 | **1.18388E-07** | Up |
| ENSG00000088766 | ***CRLS1*** | 1.18 | **0.001115548** | Up | 1.36 | **0.000185544** | Up |
| ENSG00000089335 | ***ZNF302*** |  |  |  | 1.07 | **8.00652E-07** | Up |
| ENSG00000095932 | ***SMIM24*** |  |  |  | -2.22 | **0.018673932** | Do |
| ENSG00000099624 | ***ATP5F1D*** | 1.37 | **2.19391E-09** | Up | 1.12 | **1.26432E-07** | Up |
| ENSG00000100453 | ***GZMB*** |  |  |  | -1.24 | **2.71405E-07** | Do |
| ENSG00000100532 |  |  |  |  | 1.04 | **4.1344E-11** | Up |
| ENSG00000101888 | ***NXT2*** |  |  |  | 1.04 | **5.16028E-10** | Up |
| ENSG00000102871 | ***TRADD*** | 1.04 | **4.89447E-10** | Up |  |  |  |
| ENSG00000103024 | ***NME3*** | 1.39 | **5.14694E-09** | Up | 1.33 | **7.45675E-11** | Up |
| ENSG00000103253 | ***HAGHL*** | 1.35 | **0.000667136** | Up | 1.21 | **2.41132E-07** | Up |
| ENSG00000103254 | ***ANTKMT*** | 1.81 | **1.64169E-14** | Up | 1.68 | **6.92111E-26** | Up |
| ENSG00000105327 | ***BBC3*** | 1.22 | **3.44795E-26** | Up |  |  |  |
| ENSG00000105404 | ***RABAC1*** | 1.16 | **4.0368E-09** | Up |  |  |  |
| ENSG00000105655 | ***ISYNA1*** | 1.02 | **7.32364E-06** | Up |  |  |  |
| ENSG00000106588 | ***PSMA2*** |  |  |  | 1.08 | **0.013096286** | Up |
| ENSG00000107872 | ***FBXL15*** | 2.03 | **3.08556E-17** | Up | 1.95 | **6.20909E-64** | Up |
| ENSG00000108479 | ***GALK1*** | 1.10 | **9.1627E-05** | Up |  |  |  |
| ENSG00000110628 | ***SLC22A18*** | 1.10 | **0.00013788** | Up |  |  |  |
| ENSG00000111711 | ***GOLT1B*** |  |  |  | 1.02 | **6.15069E-06** | Up |
| ENSG00000111796 | ***KLRB1*** |  |  |  | 1.01 | **0.024465459** | Up |
| ENSG00000112667 | ***DNPH1*** | 1.02 | **0.002261957** | Up | 1.02 | **2.60343E-05** | Up |
| ENSG00000113387 | ***SUB1*** |  |  |  | 1.20 | **9.32089E-05** | Up |
| ENSG00000114209 | ***PDCD10*** |  |  |  | 1.06 | **0.000253431** | Up |
| ENSG00000115514 | ***TXNDC9*** |  |  |  | 1.28 | **4.69357E-12** | Up |
| ENSG00000115540 | ***MOB4*** |  |  |  | 1.09 | **1.46176E-10** | Up |
| ENSG00000116133 | ***DHCR24*** |  |  |  | -1.13 | **0.009271137** | Do |
| ENSG00000116815 | ***CD58*** |  |  |  | 1.34 | **1.87928E-08** | Up |
| ENSG00000119669 | ***IRF2BPL*** | 1.20 | **7.12926E-11** | Up | 1.08 | **0.000814609** | Up |
| ENSG00000121350 | ***PYROXD1*** |  |  |  | 1.04 | **0.001816796** | Up |
| ENSG00000123144 | ***TRIR*** | 1.05 | **9.70918E-30** | Up |  |  |  |
| ENSG00000124074 | ***ENKD1*** | 1.01 | **0.015046952** | Up |  |  |  |
| ENSG00000125534 | ***PPDPF*** | 1.62 | **1.42232E-07** | Up | 1.14 | **0.001302395** | Up |
| ENSG00000125652 | ***ALKBH7*** | 1.31 | **1.08703E-15** | Up | 1.19 | **4.40228E-10** | Up |
| ENSG00000125910 | ***S1PR4*** | 1.12 | **1.49244E-12** | Up |  |  |  |
| ENSG00000126860 | ***EVI2A*** |  |  |  | 1.69 | **2.51388E-06** | Up |
| ENSG00000127415 | ***IDUA*** | 1.10 | **1.02482E-07** | Up | 1.11 | **1.75394E-12** | Up |
| ENSG00000127528 | ***KLF2*** | 1.66 | **3.99365E-23** | Up | 1.46 | **7.78562E-20** | Up |
| ENSG00000127995 | ***CASD1*** |  |  |  | 1.01 | **0.00026854** | Up |
| ENSG00000128011 | ***LRFN1*** | 1.58 | **4.52929E-20** | Up | 1.25 | **1.11627E-09** | Up |
| ENSG00000128228 | ***SDF2L1*** | 1.18 | **7.34503E-09** | Up |  |  |  |
| ENSG00000128708 | ***HAT1*** |  |  |  | 1.16 | **0.001213382** | Up |
| ENSG00000129757 | ***CDKN1C*** | 1.74 | **0.032920657** | Up |  |  |  |
| ENSG00000129932 | ***DOHH*** | 1.18 | **2.08501E-08** | Up | 1.03 | **1.5829E-11** | Up |
| ENSG00000129968 | ***ABHD17A*** | 1.13 | **3.92949E-05** | Up |  |  |  |
| ENSG00000130164 | ***LDLR*** |  |  |  | -1.20 | **2.07309E-21** | Do |
| ENSG00000130522 | ***JUND*** | 1.09 | **6.4365E-14** | Up |  |  |  |
| ENSG00000130748 | ***TMEM160*** | 2.85 | **1.77577E-69** | Up | 2.74 | **4.00028E-42** | Up |
| ENSG00000130881 | ***LRP3*** |  |  |  | 1.12 | **0.003110484** | Up |
| ENSG00000131401 | ***NAPSB*** |  |  |  | -1.03 | **6.04982E-05** | Do |
| ENSG00000133250 | ***ZNF414*** | 1.21 | **1.26271E-12** | Up | 1.08 | **4.62189E-11** | Up |
| ENSG00000134152 | ***KATNBL1*** |  |  |  | 1.02 | **0.000694639** | Up |
| ENSG00000135722 | ***FBXL8*** | 1.47 | **0.010988137** | Up |  |  |  |
| ENSG00000137692 | ***DCUN1D5*** |  |  |  | 1.06 | **5.29658E-06** | Up |
| ENSG00000137876 | ***RSL24D1*** |  |  |  | 1.41 | **0.003250529** | Up |
| ENSG00000138468 | ***SENP7*** |  |  |  | 1.08 | **1.29249E-09** | Up |
| ENSG00000139163 | ***ETNK1*** |  |  |  | 1.12 | **3.06609E-10** | Up |
| ENSG00000139826 | ***ABHD13*** |  |  |  | 1.14 | **3.36006E-05** | Up |
| ENSG00000140379 | ***BCL2A1*** |  |  |  | 1.47 | **0.007776025** | Up |
| ENSG00000140406 | ***TLNRD1*** | 1.30 | **1.87483E-58** | Up | 1.23 | **1.66525E-44** | Up |
| ENSG00000141933 | ***TPGS1*** | 2.86 | **2.54652E-97** | Up | 2.75 | **2.1205E-110** | Up |
| ENSG00000141965 | ***FEM1A*** | 1.16 | **1.3394E-08** | Up | 1.26 | **9.70928E-14** | Up |
| ENSG00000142409 | ***ZNF787*** | 1.35 | **2.27028E-09** | Up | 1.06 | **1.8475E-36** | Up |
| ENSG00000142544 | ***CTU1*** | 1.55 | **7.54909E-16** | Up | 1.46 | **3.787E-28** | Up |
| ENSG00000142694 | ***EVA1B*** | 2.95 | **3.19348E-30** | Up | 2.88 | **3.00605E-52** | Up |
| ENSG00000143353 | ***LYPLAL1*** |  |  |  | 1.02 | **0.000432012** | Up |
| ENSG00000143742 | ***SRP9*** |  |  |  | 1.02 | **1.51514E-07** | Up |
| ENSG00000143878 | ***RHOB*** | 1.10 | **5.80254E-08** | Up | 1.04 | **1.59381E-08** | Up |
| ENSG00000146757 | ***ZNF92*** |  |  |  | 1.13 | **7.31267E-07** | Up |
| ENSG00000147119 | ***CHST7*** | 1.71 | **0.005778502** | Up | 1.58 | **0.001296071** | Up |
| ENSG00000147813 | ***NAPRT*** | 1.55 | **2.87266E-09** | Up | 1.28 | **2.30984E-06** | Up |
| ENSG00000150045 | ***KLRF1*** | 1.11 | **0.001649658** | Up |  |  |  |
| ENSG00000151239 | ***TWF1*** |  |  |  | 1.19 | **1.60974E-14** | Up |
| ENSG00000151743 | ***AMN1*** |  |  |  | 1.30 | **4.50707E-12** | Up |
| ENSG00000152082 | ***MZT2B*** | 2.04 | **1.11953E-17** | Up | 1.91 | **9.54025E-11** | Up |
| ENSG00000152926 | ***ZNF117*** |  |  |  | 1.42 | **0.026009502** | Up |
| ENSG00000152944 | ***MED21*** |  |  |  | 1.11 | **6.36291E-10** | Up |
| ENSG00000153130 | ***SCOC*** |  |  |  | 1.25 | **0.006551535** | Up |
| ENSG00000154589 | ***LY96*** |  |  |  | 2.06 | **2.7295E-05** | Up |
| ENSG00000157107 | ***FCHO2*** |  |  |  | 1.11 | **0.000412631** | Up |
| ENSG00000158106 | ***RHPN1*** | 1.01 | **5.09166E-06** | Up |  |  |  |
| ENSG00000160256 | ***SLX9*** | 1.15 | **4.72524E-06** | Up | 1.07 | **4.93875E-06** | Up |
| ENSG00000160813 | ***PPP1R35*** | 1.02 | **1.09818E-12** | Up |  |  |  |
| ENSG00000160972 | ***PPP1R16A*** | 1.16 | **1.07249E-05** | Up | 1.07 | **7.05403E-07** | Up |
| ENSG00000161677 | ***JOSD2*** | 1.33 | **1.31958E-06** | Up | 1.12 | **3.90054E-14** | Up |
| ENSG00000162366 | ***PDZK1IP1*** |  |  |  | -1.63 | **0.013199227** | Do |
| ENSG00000162961 | ***DPY30*** |  |  |  | 1.04 | **3.07332E-07** | Up |
| ENSG00000162980 | ***ARL5A*** |  |  |  | 1.00 | **2.02927E-08** | Up |
| ENSG00000164031 | ***DNAJB14*** |  |  |  | 1.07 | **1.88263E-11** | Up |
| ENSG00000164649 | ***CDCA7L*** | -1.09 | **0.016198224** | Do |  |  |  |
| ENSG00000165169 | ***DYNLT3*** |  |  |  | 1.13 | **8.95494E-08** | Up |
| ENSG00000165804 | ***ZNF219*** | 1.70 | **7.51536E-05** | Up | 1.28 | **0.003175327** | Up |
| ENSG00000165997 | ***ARL5B*** |  |  |  | 1.01 | **7.75329E-10** | Up |
| ENSG00000166200 | ***COPS2*** |  |  |  | 1.08 | **3.42381E-06** | Up |
| ENSG00000166479 | ***TMX3*** |  |  |  | 1.00 | **6.27167E-07** | Up |
| ENSG00000167766 | ***ZNF83*** |  |  |  | 1.33 | **1.17648E-05** | Up |
| ENSG00000168282 | ***MGAT2*** | 1.38 | **2.60489E-05** | Up | 1.39 | **2.61037E-06** | Up |
| ENSG00000169019 | ***COMMD8*** | 1.55 | **0.026275244** | Up | 1.81 | **1.83969E-10** | Up |
| ENSG00000170385 | ***SLC30A1*** |  |  |  | 1.14 | **4.17134E-08** | Up |
| ENSG00000170604 | ***IRF2BP1*** | 1.08 | **1.435E-10** | Up |  |  |  |
| ENSG00000170638 | ***TRABD*** | 1.07 | **1.58698E-17** | Up |  |  |  |
| ENSG00000170855 | ***TRIAP1*** |  |  |  | 1.08 | **0.000934995** | Up |
| ENSG00000171204 | ***TMEM126B*** |  |  |  | 1.27 | **2.42331E-05** | Up |
| ENSG00000171222 | ***SCAND1*** | 1.57 | **7.65707E-13** | Up | 1.31 | **1.84152E-77** | Up |
| ENSG00000171223 | ***JUNB*** | 1.36 | **1.66115E-05** | Up | 1.24 | **0.00025359** | Up |
| ENSG00000171443 | ***ZNF524*** | 1.13 | **1.16092E-06** | Up |  |  |  |
| ENSG00000172062 | ***SMN1*** | -2.06 | **4.84429E-18** | Do | -1.58 | **6.34224E-11** | Do |
| ENSG00000172216 | ***CEBPB*** | 1.89 | **1.89475E-13** | Up | 1.64 | **3.4869E-10** | Up |
| ENSG00000173198 | ***CYSLTR1*** |  |  |  | 1.04 | **0.011949606** | Up |
| ENSG00000173272 | ***MZT2A*** | 1.20 | **3.89616E-05** | Up | 1.21 | **1.69294E-07** | Up |
| ENSG00000173621 | ***LRFN4*** | 1.33 | **8.10221E-07** | Up | 1.33 | **1.52922E-05** | Up |
| ENSG00000174946 | ***GPR171*** |  |  |  | 1.11 | **0.00414035** | Up |
| ENSG00000175602 | ***CCDC85B*** | 2.87 | **5.09013E-21** | Up | 2.72 | **1.15305E-58** | Up |
| ENSG00000176386 | ***CDC26*** |  |  |  | -1.03 | **8.99708E-07** | Do |
| ENSG00000176973 | ***FAM89B*** | 1.01 | **1.15493E-07** | Up |  |  |  |
| ENSG00000177888 | ***ZBTB41*** |  |  |  | 1.09 | **1.31038E-20** | Up |
| ENSG00000177989 | ***ODF3B*** | 1.44 | **0.004781429** | Up | 1.67 | **0.0001691** | Up |
| ENSG00000178075 | ***GRAMD1C*** | 1.34 | **0.004072694** | Up | 1.57 | **2.70549E-05** | Up |
| ENSG00000178464 | ***RPL10P16*** | 1.40 | **8.87363E-14** | Up | 1.32 | **0.000357188** | Up |
| ENSG00000179304 | ***FAM156B*** |  |  |  | -2.00 | **1.22596E-05** | Do |
| ENSG00000179588 | ***ZFPM1*** |  |  |  | 1.23 | **1.02428E-08** | Up |
| ENSG00000179862 | ***CITED4*** | 1.59 | **7.0912E-08** | Up | 1.47 | **0.000293448** | Up |
| ENSG00000179941 | ***BBS10*** |  |  |  | 1.15 | **0.000462489** | Up |
| ENSG00000179978 | ***NAIPP2*** | -1.60 | **0.001674627** | Do |  |  |  |
| ENSG00000180233 | ***ZNRF2*** | 1.03 | **1.04139E-05** | Up | 1.36 | **2.03253E-21** | Up |
| ENSG00000181029 | ***TRAPPC5*** | 1.99 | **0.000574423** | Up | 1.68 | **3.55545E-13** | Up |
| ENSG00000181061 | ***HIGD1A*** |  |  |  | 1.30 | **4.63395E-07** | Up |
| ENSG00000181444 | ***ZNF467*** | 1.59 | **0.000122306** | Up |  |  |  |
| ENSG00000182087 | ***TMEM259*** | 1.03 | **1.40363E-21** | Up |  |  |  |
| ENSG00000182154 | ***MRPL41*** | 1.11 | **1.392E-06** | Up |  |  |  |
| ENSG00000182287 | ***AP1S2*** |  |  |  | 1.19 | **8.09123E-10** | Up |
| ENSG00000182809 | ***CRIP2*** | 1.30 | **0.044632487** | Up |  |  |  |
| ENSG00000182871 | ***COL18A1*** |  |  |  | 1.15 | **0.013973787** | Up |
| ENSG00000183458 | ***PKD1P3*** |  |  |  | 1.45 | **2.84379E-13** | Up |
| ENSG00000183779 | ***ZNF703*** | 1.39 | **0.017747016** | Up | 1.07 | **0.000176114** | Up |
| ENSG00000184221 | ***OLIG1*** |  |  |  | 1.15 | **0.032586055** | Up |
| ENSG00000184897 | ***H1-10*** | 1.85 | **2.04334E-21** | Up | 1.48 | **6.67063E-34** | Up |
| ENSG00000185436 | ***IFNLR1*** | -1.44 | **0.000467383** | Do | -1.11 | **2.20285E-05** | Do |
| ENSG00000185947 | ***ZNF267*** |  |  |  | 1.12 | **9.36468E-09** | Up |
| ENSG00000186395 | ***KRT10*** | 1.22 | **7.93854E-08** | Up | 1.19 | **3.76674E-13** | Up |
| ENSG00000186827 | ***TNFRSF4*** | 1.72 | **0.000784254** | Up | 1.52 | **2.01903E-06** | Up |
| ENSG00000187922 | ***LCN10*** | -1.19 | **0.003737614** | Do |  |  |  |
| ENSG00000188483 | ***IER5L*** | 3.26 | **6.90835E-14** | Up | 2.92 | **1.08975E-10** | Up |
| ENSG00000188820 | ***CALHM6*** |  |  |  | 1.46 | **0.002649043** | Up |
| ENSG00000196126 | ***HLA-DRB1*** | -1.02 | **0.000162278** | Do | -1.04 | **0.005403963** | Do |
| ENSG00000196268 | ***ZNF493*** |  |  |  | 1.04 | **0.003774737** | Up |
| ENSG00000196358 | ***NTNG2*** | 1.28 | **4.35515E-07** | Up | 1.29 | **0.000526614** | Up |
| ENSG00000196911 | ***KPNA5*** |  |  |  | 1.07 | **0.006141479** | Up |
| ENSG00000197044 | ***ZNF441*** |  |  |  | 1.11 | **0.000194343** | Up |
| ENSG00000197045 | ***GMFB*** |  |  |  | 1.18 | **8.12026E-06** | Up |
| ENSG00000197056 | ***ZMYM1*** |  |  |  | 1.02 | **1.69906E-05** | Up |
| ENSG00000197182 | ***MIRLET7BHG*** | 1.20 | **0.000758972** | Up | 1.03 | **0.000276537** | Up |
| ENSG00000197483 | ***ZNF628*** | 1.91 | **2.29969E-17** | Up |  |  |  |
| ENSG00000197530 | ***MIB2*** | 1.23 | **7.46385E-05** | Up |  |  |  |
| ENSG00000197530 | ***MIB2*** |  |  |  | 1.02 | **8.72144E-08** | Up |
| ENSG00000197982 | ***C1orf122*** | 1.27 | **1.32331E-12** | Up | 1.13 | **1.74663E-15** | Up |
| ENSG00000198355 | ***PIM3*** | 1.11 | **0.029320954** | Up | 1.07 | **6.69837E-05** | Up |
| ENSG00000198712 | ***MT-CO2*** |  |  |  | -1.01 | **2.35908E-14** | Do |
| ENSG00000198727 | ***MT-CYB*** |  |  |  | -1.03 | **2.50631E-05** | Do |
| ENSG00000198786 | ***MT-ND5*** |  |  |  | -1.04 | **0.005981194** | Do |
| ENSG00000198886 | ***MT-ND4*** |  |  |  | -1.09 | **2.58305E-07** | Do |
| ENSG00000198898 | ***CAPZA2*** |  |  |  | 1.29 | **3.12963E-09** | Up |
| ENSG00000198899 | ***MT-ATP6*** |  |  |  | -1.03 | **1.94828E-10** | Do |
| ENSG00000203644 |  | 2.18 | **8.78422E-07** | Up | 2.01 | **1.25391E-08** | Up |
| ENSG00000203896 | ***LIME1*** | 1.46 | **6.82499E-07** | Up | 1.23 | **9.99245E-08** | Up |
| ENSG00000204475 | ***NCR3*** |  |  |  | -1.02 | **5.18488E-05** | Do |
| ENSG00000204839 | ***MROH6*** | 1.11 | **0.008771969** | Up |  |  |  |
| ENSG00000205571 | ***SMN2*** | 1.10 | **0.016448202** | Up | 1.25 | **0.001127368** | Up |
| ENSG00000205609 | ***EIF3CL*** | -1.86 | **1.20341E-05** | Do | -1.67 | **2.03998E-10** | Do |
| ENSG00000211459 | ***MT-RNR1*** |  |  |  | -1.04 | **1.95244E-05** | Do |
| ENSG00000213347 | ***MXD3*** |  |  |  | 1.04 | **0.001037915** | Up |
| ENSG00000213442 | ***RPL18AP3*** | 1.83 | **1.33555E-11** | Up | 1.71 | **1.26722E-12** | Up |
| ENSG00000213563 | ***C8orf82*** | 1.08 | **1.48147E-06** | Up |  |  |  |
| ENSG00000215472 | ***RPL17-C18orf32*** | 2.72 | **0.000227745** | Up | 2.77 | **5.53835E-07** | Up |
| ENSG00000218891 | ***ZNF579*** | 1.94 | **5.00996E-16** | Up |  |  |  |
| ENSG00000219410 |  | 1.95 | **6.21489E-08** | Up | 1.42 | **0.028224593** | Up |
| ENSG00000220008 | ***LINGO3*** | 2.27 | **1.12908E-23** | Up | 2.38 | **8.84942E-27** | Up |
| ENSG00000220793 | ***RPL21P119*** | 4.81 | **7.86167E-09** | Up | 5.01 | **8.79094E-07** | Up |
| ENSG00000220842 | ***RPL21P16*** | 2.65 | **0.004770874** | Up | 2.61 | **4.20004E-13** | Up |
| ENSG00000221869 | ***CEBPD*** | 3.07 | **1.12171E-22** | Up | 2.78 | **1.00082E-25** | Up |
| ENSG00000225339 |  |  |  |  | -1.29 | **5.07447E-24** | Do |
| ENSG00000225342 | ***LRRK2-DT*** |  |  |  | 1.39 | **0.003877205** | Up |
| ENSG00000227671 | ***ZNF731P*** |  |  |  | 1.24 | **0.005634723** | Up |
| ENSG00000231259 | ***ANAPC1P2*** |  |  |  | -1.86 | **0.001233676** | Do |
| ENSG00000232573 | ***RPL3P4*** | 1.42 | **4.00064E-06** | Up | 1.24 | **0.006171174** | Up |
| ENSG00000233429 | ***HOTAIRM1*** | 1.66 | **0.01495329** | Up | 1.35 | **0.000343129** | Up |
| ENSG00000233476 | ***EEF1A1P6*** | 1.97 | **1.32695E-69** | Up | 2.05 | **8.75575E-05** | Up |
| ENSG00000234797 | ***RPS3AP6*** |  |  |  | 2.68 | **9.24165E-05** | Up |
| ENSG00000236552 | ***RPL13AP5*** | 1.32 | **2.84628E-06** | Up | 1.25 | **7.17772E-07** | Up |
| ENSG00000237973 | ***MTCO1P12*** | -1.24 | **0.040296366** | Do | -1.65 | **6.87269E-07** | Do |
| ENSG00000239775 |  |  |  |  | 1.31 | **0.000557627** | Up |
| ENSG00000240972 | ***MIF*** | 1.09 | **1.53503E-07** | Up |  |  |  |
| ENSG00000242539 |  | 1.04 | **0.003301345** | Up | 1.08 | **7.01195E-06** | Up |
| ENSG00000242616 | ***GNG10*** |  |  |  | 1.45 | **0.000115204** | Up |
| ENSG00000243414 | ***TICAM2*** |  |  |  | 1.15 | **0.003982302** | Up |
| ENSG00000243679 |  | 1.28 | **0.000635777** | Up |  |  |  |
| ENSG00000251201 | ***TMED7-TICAM2*** | -1.29 | **0.000242708** | Do |  |  |  |
| ENSG00000254413 | ***CHKB-CPT1B*** |  |  |  | 1.08 | **0.022121264** | Up |
| ENSG00000254685 | ***FPGT*** |  |  |  | 1.27 | **1.76424E-07** | Up |
| ENSG00000254692 |  | -6.12 | **1.01186E-09** | Do | -5.37 | **8.61052E-12** | Do |
| ENSG00000254873 |  | 2.10 | **1.08782E-06** | Up | 1.89 | **7.41066E-05** | Up |
| ENSG00000257704 | ***INAFM1*** | 1.22 | **5.56433E-10** | Up |  |  |  |
| ENSG00000257764 |  | 4.07 | **1.62657E-12** | Up | 3.90 | **1.03906E-07** | Up |
| ENSG00000258377 |  | -9.72 | **1.15831E-44** | Do | -6.65 | **3.00434E-33** | Do |
| ENSG00000259132 |  |  |  |  | -1.88 | **0.005606333** | Do |
| ENSG00000259399 | ***TGIF2-RAB5IF*** | -6.24 | **1.55372E-13** | Do | -5.93 | **7.09655E-12** | Do |
| ENSG00000260772 |  | 1.47 | **8.65824E-12** | Up | 1.24 | **4.85002E-08** | Up |
| ENSG00000261221 | ***ZNF865*** | 1.44 | **2.00014E-25** | Up | 1.12 | **4.77203E-28** | Up |
| ENSG00000261796 | ***ISY1-RAB43*** | -1.11 | **1.80099E-10** | Do | -1.07 | **3.01686E-09** | Do |
| ENSG00000263244 |  |  |  |  | 1.27 | **0.001042377** | Up |
| ENSG00000265666 | ***RARA-AS1*** | 1.15 | **0.008065409** | Up |  |  |  |
| ENSG00000266642 |  | -1.74 | **5.16215E-07** | Do |  |  |  |
| ENSG00000268350 | ***FAM156A*** |  |  |  | 1.41 | **0.031814041** | Up |
| ENSG00000269378 | ***ITGB1P1*** | -1.22 | **0.033470882** | Do | -1.39 | **0.005774784** | Do |
| ENSG00000269955 | ***FMC1-LUC7L2*** | -1.78 | **2.45048E-10** | Do | -1.73 | **5.62436E-09** | Do |
| ENSG00000271533 |  |  |  |  | 1.13 | **1.47686E-07** | Up |
| ENSG00000272410 |  | -2.00 | **2.58668E-13** | Do | -1.24 | **0.002206342** | Do |
| ENSG00000272449 |  | 1.99 | **1.80244E-16** | Up | 1.83 | **3.74914E-17** | Up |
| ENSG00000274012 | ***RN7SL2*** | 2.61 | **0.022170491** | Up | 2.25 | **0.022505083** | Up |
| ENSG00000274049 | ***INO80B-WBP1*** | -2.24 | **0.000112652** | Do | -2.54 | **0.001311019** | Do |
| ENSG00000274272 |  | 1.61 | **7.78288E-14** | Up | 1.39 | **4.60866E-09** | Up |
| ENSG00000274425 |  | 1.90 | **2.65319E-26** | Up | 1.69 | **0.001857291** | Up |
| ENSG00000275764 |  |  |  |  | 1.12 | **9.32704E-05** | Up |
| ENSG00000276168 | ***RN7SL1*** | 2.17 | **0.000284169** | Up | 1.96 | **0.017710642** | Up |
| ENSG00000277053 | ***GTF2IP1*** | -8.82 | **2.31621E-06** | Do | -8.75 | **5.56698E-06** | Do |
| ENSG00000279364 |  | 1.30 | **2.37678E-05** | Up |  |  |  |
| ENSG00000279753 |  |  |  |  | -1.35 | **1.32044E-08** | Do |
| ENSG00000280088 |  | 1.09 | **0.025716097** | Up |  |  |  |
| ENSG00000280734 | ***LINC01232*** | -1.03 | **0.045443269** | Do |  |  |  |
| ENSG00000283041 |  | 1.91 | **6.8829E-22** | Up | 1.89 | **6.38804E-15** | Up |
| ENSG00000283839 |  | 1.84 | **2.23574E-08** | Up | 1.76 | **6.40652E-09** | Up |
| ENSG00000284930 |  |  |  |  | 1.46 | **0.010516622** | Up |
| ENSG00000284976 |  | 2.01 | **0.001482281** | Up | 1.93 | **0.035684783** | Up |
| ENSG00000285304 |  | -1.25 | **0.000207904** | Do | -1.15 | **0.028707226** | Do |
| ENSG00000286129 |  | -1.20 | **0.013802835** | Do |  |  |  |
| ENSG00000286140 | ***DERPC*** |  |  |  | -1.04 | **0.018228068** | Do |
| ENSG00000286190 |  | 1.71 | **0.000758407** | Up |  |  |  |
| ENSG00000289701 |  | 1.14 | **0.042193062** | Up |  |  |  |
| ENSG00000290018 |  | 5.77 | **7.3001E-22** | Up | 5.35 | **3.78336E-32** | Up |
| ENSG00000290038 |  | -9.62 | **4.1154E-50** | Do | -7.44 | **4.66105E-40** | Do |
| ENSG00000291103 | ***MPHOSPH10P1*** |  |  |  | 1.70 | **6.4853E-06** | Up |
| ENSG00000291152 | ***SBDSP1*** |  |  |  | 1.14 | **0.00052124** | Up |
| ENSG00000291230 |  | 1.47 | **4.16965E-05** | Up | 1.50 | **6.5958E-07** | Up |
| ENSG00000291316 |  |  |  |  | -1.75 | **2.26175E-19** | Do |

***BIS_Table:***

|  |  | **log2FC** | **FDR** | **SMA T0 vs HC** | **log2FC** | **FDR** | **SMA T10 vs HC** |
| --- | --- | --- | --- | --- | --- | --- | --- |
| ENSG00000006015 | ***REX1BD*** | 1.45 | **1.25172E-23** | Up | 1.24 | **3.18041E-40** | Up |
| ENSG00000007520 | ***TSR3*** | 1.02 | **2.934E-10** | Up |  |  |  |
| ENSG00000025708 | ***TYMP*** | 1.22 | **0.001129937** | Up |  |  |  |
| ENSG00000070423 | ***RNF126*** | 1.00 | **2.83459E-12** | Up |  |  |  |
| ENSG00000073150 | ***PANX2*** | 1.49 | **8.53629E-05** | Up | 1.10 | **0.012991063** | Up |
| ENSG00000088766 | ***CRLS1*** | 1.18 | **0.001115548** | Up | 1.36 | **0.000185544** | Up |
| ENSG00000099624 | ***ATP5F1D*** | 1.37 | **2.19391E-09** | Up | 1.12 | **1.26432E-07** | Up |
| ENSG00000102871 | ***TRADD*** | 1.04 | **4.89447E-10** | Up |  |  |  |
| ENSG00000103024 | ***NME3*** | 1.39 | **5.14694E-09** | Up | 1.33 | **7.45675E-11** | Up |
| ENSG00000103253 | ***HAGHL*** | 1.35 | **0.000667136** | Up | 1.21 | **2.41132E-07** | Up |
| ENSG00000103254 | ***ANTKMT*** | 1.81 | **1.64169E-14** | Up | 1.68 | **6.92111E-26** | Up |
| ENSG00000105327 | ***BBC3*** | 1.22 | **3.44795E-26** | Up |  |  |  |
| ENSG00000105404 | ***RABAC1*** | 1.16 | **4.0368E-09** | Up |  |  |  |
| ENSG00000105655 | ***ISYNA1*** | 1.02 | **7.32364E-06** | Up |  |  |  |
| ENSG00000107872 | ***FBXL15*** | 2.03 | **3.08556E-17** | Up | 1.95 | **6.20909E-64** | Up |
| ENSG00000108479 | ***GALK1*** | 1.10 | **9.1627E-05** | Up |  |  |  |
| ENSG00000110628 | ***SLC22A18*** | 1.10 | **0.00013788** | Up |  |  |  |
| ENSG00000112667 | ***DNPH1*** | 1.02 | **0.002261957** | Up | 1.02 | **2.60343E-05** | Up |
| ENSG00000119669 | ***IRF2BPL*** | 1.20 | **7.12926E-11** | Up | 1.08 | **0.000814609** | Up |
| ENSG00000123144 | ***TRIR*** | 1.05 | **9.70918E-30** | Up |  |  |  |
| ENSG00000124074 | ***ENKD1*** | 1.01 | **0.015046952** | Up |  |  |  |
| ENSG00000125534 | ***PPDPF*** | 1.62 | **1.42232E-07** | Up | 1.14 | **0.001302395** | Up |
| ENSG00000125652 | ***ALKBH7*** | 1.31 | **1.08703E-15** | Up | 1.19 | **4.40228E-10** | Up |
| ENSG00000125910 | ***S1PR4*** | 1.12 | **1.49244E-12** | Up |  |  |  |
| ENSG00000127415 | ***IDUA*** | 1.10 | **1.02482E-07** | Up | 1.11 | **1.75394E-12** | Up |
| ENSG00000127528 | ***KLF2*** | 1.66 | **3.99365E-23** | Up | 1.46 | **7.78562E-20** | Up |
| ENSG00000128011 | ***LRFN1*** | 1.58 | **4.52929E-20** | Up | 1.25 | **1.11627E-09** | Up |
| ENSG00000128228 | ***SDF2L1*** | 1.18 | **7.34503E-09** | Up |  |  |  |
| ENSG00000129757 | ***CDKN1C*** | 1.74 | **0.032920657** | Up |  |  |  |
| ENSG00000129932 | ***DOHH*** | 1.18 | **2.08501E-08** | Up | 1.03 | **1.5829E-11** | Up |
| ENSG00000129968 | ***ABHD17A*** | 1.13 | **3.92949E-05** | Up |  |  |  |
| ENSG00000130522 | ***JUND*** | 1.09 | **6.4365E-14** | Up |  |  |  |
| ENSG00000130748 | ***TMEM160*** | 2.85 | **1.77577E-69** | Up | 2.74 | **4.00028E-42** | Up |
| ENSG00000133250 | ***ZNF414*** | 1.21 | **1.26271E-12** | Up | 1.08 | **4.62189E-11** | Up |
| ENSG00000135722 | ***FBXL8*** | 1.47 | **0.010988137** | Up |  |  |  |
| ENSG00000140406 | ***TLNRD1*** | 1.30 | **1.87483E-58** | Up | 1.23 | **1.66525E-44** | Up |
| ENSG00000141933 | ***TPGS1*** | 2.86 | **2.54652E-97** | Up | 2.75 | **2.1205E-110** | Up |
| ENSG00000141965 | ***FEM1A*** | 1.16 | **1.3394E-08** | Up | 1.26 | **9.70928E-14** | Up |
| ENSG00000142409 | ***ZNF787*** | 1.35 | **2.27028E-09** | Up | 1.06 | **1.8475E-36** | Up |
| ENSG00000142544 | ***CTU1*** | 1.55 | **7.54909E-16** | Up | 1.46 | **3.787E-28** | Up |
| ENSG00000142694 | ***EVA1B*** | 2.95 | **3.19348E-30** | Up | 2.88 | **3.00605E-52** | Up |
| ENSG00000143878 | ***RHOB*** | 1.10 | **5.80254E-08** | Up | 1.04 | **1.59381E-08** | Up |
| ENSG00000147119 | ***CHST7*** | 1.71 | **0.005778502** | Up | 1.58 | **0.001296071** | Up |
| ENSG00000147813 | ***NAPRT*** | 1.55 | **2.87266E-09** | Up | 1.28 | **2.30984E-06** | Up |
| ENSG00000150045 | ***KLRF1*** | 1.11 | **0.001649658** | Up |  |  |  |
| ENSG00000152082 | ***MZT2B*** | 2.04 | **1.11953E-17** | Up | 1.91 | **9.54025E-11** | Up |
| ENSG00000158106 | ***RHPN1*** | 1.01 | **5.09166E-06** | Up |  |  |  |
| ENSG00000160256 | ***SLX9*** | 1.15 | **4.72524E-06** | Up | 1.07 | **4.93875E-06** | Up |
| ENSG00000160813 | ***PPP1R35*** | 1.02 | **1.09818E-12** | Up |  |  |  |
| ENSG00000160972 | ***PPP1R16A*** | 1.16 | **1.07249E-05** | Up | 1.07 | **7.05403E-07** | Up |
| ENSG00000161677 | ***JOSD2*** | 1.33 | **1.31958E-06** | Up | 1.12 | **3.90054E-14** | Up |
| ENSG00000164649 | ***CDCA7L*** | -1.09 | **0.016198224** | Do |  |  |  |
| ENSG00000165804 | ***ZNF219*** | 1.70 | **7.51536E-05** | Up | 1.28 | **0.003175327** | Up |
| ENSG00000168282 | ***MGAT2*** | 1.38 | **2.60489E-05** | Up | 1.39 | **2.61037E-06** | Up |
| ENSG00000169019 | ***COMMD8*** | 1.55 | **0.026275244** | Up | 1.81 | **1.83969E-10** | Up |
| ENSG00000170604 | ***IRF2BP1*** | 1.08 | **1.435E-10** | Up |  |  |  |
| ENSG00000170638 | ***TRABD*** | 1.07 | **1.58698E-17** | Up |  |  |  |
| ENSG00000171222 | ***SCAND1*** | 1.57 | **7.65707E-13** | Up | 1.31 | **1.84152E-77** | Up |
| ENSG00000171223 | ***JUNB*** | 1.36 | **1.66115E-05** | Up | 1.24 | **0.00025359** | Up |
| ENSG00000171443 | ***ZNF524*** | 1.13 | **1.16092E-06** | Up |  |  |  |
| ENSG00000172062 | ***SMN1*** | -2.06 | **4.84429E-18** | Do | -1.58 | **6.34224E-11** | Do |
| ENSG00000172216 | ***CEBPB*** | 1.89 | **1.89475E-13** | Up | 1.64 | **3.4869E-10** | Up |
| ENSG00000173272 | ***MZT2A*** | 1.20 | **3.89616E-05** | Up | 1.21 | **1.69294E-07** | Up |
| ENSG00000173621 | ***LRFN4*** | 1.33 | **8.10221E-07** | Up | 1.33 | **1.52922E-05** | Up |
| ENSG00000175602 | ***CCDC85B*** | 2.87 | **5.09013E-21** | Up | 2.72 | **1.15305E-58** | Up |
| ENSG00000176973 | ***FAM89B*** | 1.01 | **1.15493E-07** | Up |  |  |  |
| ENSG00000177989 | ***ODF3B*** | 1.44 | **0.004781429** | Up | 1.67 | **0.0001691** | Up |
| ENSG00000178075 | ***GRAMD1C*** | 1.34 | **0.004072694** | Up | 1.57 | **2.70549E-05** | Up |
| ENSG00000178464 | ***RPL10P16*** | 1.40 | **8.87363E-14** | Up | 1.32 | **0.000357188** | Up |
| ENSG00000179862 | ***CITED4*** | 1.59 | **7.0912E-08** | Up | 1.47 | **0.000293448** | Up |
| ENSG00000179978 | ***NAIPP2*** | -1.60 | **0.001674627** | Do |  |  |  |
| ENSG00000180233 | ***ZNRF2*** | 1.03 | **1.04139E-05** | Up | 1.36 | **2.03253E-21** | Up |
| ENSG00000181029 | ***TRAPPC5*** | 1.99 | **0.000574423** | Up | 1.68 | **3.55545E-13** | Up |
| ENSG00000181444 | ***ZNF467*** | 1.59 | **0.000122306** | Up |  |  |  |
| ENSG00000182087 | ***TMEM259*** | 1.03 | **1.40363E-21** | Up |  |  |  |
| ENSG00000182154 | ***MRPL41*** | 1.11 | **1.392E-06** | Up |  |  |  |
| ENSG00000182809 | ***CRIP2*** | 1.30 | **0.044632487** | Up |  |  |  |
| ENSG00000183779 | ***ZNF703*** | 1.39 | **0.017747016** | Up | 1.07 | **0.000176114** | Up |
| ENSG00000184897 | ***H1-10*** | 1.85 | **2.04334E-21** | Up | 1.48 | **6.67063E-34** | Up |
| ENSG00000185436 | ***IFNLR1*** | -1.44 | **0.000467383** | Do | -1.11 | **2.20285E-05** | Do |
| ENSG00000186395 | ***KRT10*** | 1.22 | **7.93854E-08** | Up | 1.19 | **3.76674E-13** | Up |
| ENSG00000186827 | ***TNFRSF4*** | 1.72 | **0.000784254** | Up | 1.52 | **2.01903E-06** | Up |
| ENSG00000187922 | ***LCN10*** | -1.19 | **0.003737614** | Do |  |  |  |
| ENSG00000188483 | ***IER5L*** | 3.26 | **6.90835E-14** | Up | 2.92 | **1.08975E-10** | Up |
| ENSG00000196126 | ***HLA-DRB1*** | -1.02 | **0.000162278** | Do | -1.04 | **0.005403963** | Do |
| ENSG00000196358 | ***NTNG2*** | 1.28 | **4.35515E-07** | Up | 1.29 | **0.000526614** | Up |
| ENSG00000197182 | ***MIRLET7BHG*** | 1.20 | **0.000758972** | Up | 1.03 | **0.000276537** | Up |
| ENSG00000197483 | ***ZNF628*** | 1.91 | **2.29969E-17** | Up |  |  |  |
| ENSG00000197530 | ***MIB2*** | 1.23 | **7.46385E-05** | Up |  |  |  |
| ENSG00000197982 | ***C1orf122*** | 1.27 | **1.32331E-12** | Up | 1.13 | **1.74663E-15** | Up |
| ENSG00000198355 | ***PIM3*** | 1.11 | **0.029320954** | Up | 1.07 | **6.69837E-05** | Up |
| ENSG00000203896 | ***LIME1*** | 1.46 | **6.82499E-07** | Up | 1.23 | **9.99245E-08** | Up |
| ENSG00000204839 | ***MROH6*** | 1.11 | **0.008771969** | Up |  |  |  |
| ENSG00000205571 | ***SMN2*** | 1.10 | **0.016448202** | Up | 1.25 | **0.001127368** | Up |
| ENSG00000205609 | ***EIF3CL*** | -1.86 | **1.20341E-05** | Do | -1.67 | **2.03998E-10** | Do |
| ENSG00000213442 | ***RPL18AP3*** | 1.83 | **1.33555E-11** | Up | 1.71 | **1.26722E-12** | Up |
| ENSG00000213563 | ***C8orf82*** | 1.08 | **1.48147E-06** | Up |  |  |  |
| ENSG00000215472 | ***RPL17-C18orf32*** | 2.72 | **0.000227745** | Up | 2.77 | **5.53835E-07** | Up |
| ENSG00000218891 | ***ZNF579*** | 1.94 | **5.00996E-16** | Up |  |  |  |
| ENSG00000220008 | ***LINGO3*** | 2.27 | **1.12908E-23** | Up | 2.38 | **8.84942E-27** | Up |
| ENSG00000220793 | ***RPL21P119*** | 4.81 | **7.86167E-09** | Up | 5.01 | **8.79094E-07** | Up |
| ENSG00000220842 | ***RPL21P16*** | 2.65 | **0.004770874** | Up | 2.61 | **4.20004E-13** | Up |
| ENSG00000221869 | ***CEBPD*** | 3.07 | **1.12171E-22** | Up | 2.78 | **1.00082E-25** | Up |
| ENSG00000232573 | ***RPL3P4*** | 1.42 | **4.00064E-06** | Up | 1.24 | **0.006171174** | Up |
| ENSG00000233429 | ***HOTAIRM1*** | 1.66 | **0.01495329** | Up | 1.35 | **0.000343129** | Up |
| ENSG00000233476 | ***EEF1A1P6*** | 1.97 | **1.32695E-69** | Up | 2.05 | **8.75575E-05** | Up |
| ENSG00000236552 | ***RPL13AP5*** | 1.32 | **2.84628E-06** | Up | 1.25 | **7.17772E-07** | Up |
| ENSG00000237973 | ***MTCO1P12*** | -1.24 | **0.040296366** | Do | -1.65 | **6.87269E-07** | Do |
| ENSG00000240972 | ***MIF*** | 1.09 | **1.53503E-07** | Up |  |  |  |
| ENSG00000251201 | ***TMED7-TICAM2*** | -1.29 | **0.000242708** | Do |  |  |  |
| ENSG00000257704 | ***INAFM1*** | 1.22 | **5.56433E-10** | Up |  |  |  |
| ENSG00000259399 | ***TGIF2-RAB5IF*** | -6.24 | **1.55372E-13** | Do | -5.93 | **7.09655E-12** | Do |
| ENSG00000261221 | ***ZNF865*** | 1.44 | **2.00014E-25** | Up | 1.12 | **4.77203E-28** | Up |
| ENSG00000261796 | ***ISY1-RAB43*** | -1.11 | **1.80099E-10** | Do | -1.07 | **3.01686E-09** | Do |
| ENSG00000265666 | ***RARA-AS1*** | 1.15 | **0.008065409** | Up |  |  |  |
| ENSG00000269378 | ***ITGB1P1*** | -1.22 | **0.033470882** | Do | -1.39 | **0.005774784** | Do |
| ENSG00000269955 | ***FMC1-LUC7L2*** | -1.78 | **2.45048E-10** | Do | -1.73 | **5.62436E-09** | Do |
| ENSG00000274012 | ***RN7SL2*** | 2.61 | **0.022170491** | Up | 2.25 | **0.022505083** | Up |
| ENSG00000274049 | ***INO80B-WBP1*** | -2.24 | **0.000112652** | Do | -2.54 | **0.001311019** | Do |
| ENSG00000276168 | ***RN7SL1*** | 2.17 | **0.000284169** | Up | 1.96 | **0.017710642** | Up |
| ENSG00000277053 | ***GTF2IP1*** | -8.82 | **2.31621E-06** | Do | -8.75 | **5.56698E-06** | Do |
| ENSG00000280734 | ***LINC01232*** | -1.03 | **0.045443269** | Do |  |  |  |

***Supplementary-Table3:*** RNAs resulted differentially expressed from individual T10 *v*s T0 comparisons in the SMA patients (6 available longitudinal samples).

**SMA_01**

| **ENS_ID** | ***Gene Symbol*** | **log2FC** | **FDR** | **SMA_T10 vs T0** |
| --- | --- | --- | --- | --- |
| ENSG00000290038 |  | -6.60 | **1.31997E-32** | Do |
| ENSG00000179304 | ***FAM156B*** | -5.34 | **1.62857E-35** | Do |
| ENSG00000205609 | ***EIF3CL*** | -1.07 | **0.003524435** | Do |
| ENSG00000275895 |  | -6.76 | **4.97529E-36** | Do |
| ENSG00000219410 |  | -2.36 | **2.64512E-10** | Do |
| ENSG00000291316 |  | -1.29 | **0.002408299** | Do |
| ENSG00000259132 |  | -1.95 | **0.006783433** | Do |
| ENSG00000211895 | ***IGHA1*** | -1.17 | **1.12782E-05** | Do |
| ENSG00000211892 | ***IGHG4*** | -1.29 | **0.013337874** | Do |
| ENSG00000257767 |  | -2.05 | **6.99483E-05** | Do |
| ENSG00000205572 | ***SERF1B*** | -1.92 | **2.076E-05** | Do |
| ENSG00000206047 | ***DEFA1*** | 7.49 | **3.79505E-55** | Up |
| ENSG00000224186 | ***PITX1-AS1*** | 2.24 | **2.40527E-06** | Up |
| ENSG00000272980 |  | 1.69 | **0.017250972** | Up |
| ENSG00000249624 |  | 1.58 | **1.91911E-06** | Up |
| ENSG00000265118 |  | 1.37 | **0.000703955** | Up |
| ENSG00000172058 | ***SERF1A*** | 3.72 | **3.53173E-13** | Up |
| ENSG00000183604 | ***SMG1P5*** | 1.63 | **0.028272067** | Up |
| ENSG00000179348 | ***GATA2*** | 1.08 | **0.040903896** | Up |

**SMA_02**

| **ENS_ID** | ***Gene Symbol*** | **log2FC** | **FDR** | **SMA_T10 vs T0** |
| --- | --- | --- | --- | --- |
| ENSG00000275895 |  | -6.90 | **3.12112E-32** | Do |
| ENSG00000291316 |  | -2.47 | **6.97038E-08** | Do |
| ENSG00000206047 | ***DEFA1*** | -5.78 | **4.61057E-16** | Do |
| ENSG00000279753 |  | -1.63 | **0.006916451** | Do |
| ENSG00000286185 |  | -7.00 | **1.5528E-30** | Do |
| ENSG00000267645 |  | -2.40 | **4.73144E-06** | Do |
| ENSG00000180644 | ***PRF1*** | -1.45 | **0.044418555** | Do |
| ENSG00000224186 | ***PITX1-AS1*** | 3.61 | **1.80979E-08** | Up |
| ENSG00000290683 |  | 2.06 | **0.038113651** | Up |
| ENSG00000280987 | ***MATR3*** | 2.85 | **8.86492E-08** | Up |
| ENSG00000133742 | ***CA1*** | 1.67 | **0.011736672** | Up |
| ENSG00000266820 | ***KPNA2P3*** | 2.26 | **0.00274903** | Up |
| ENSG00000214425 | ***LRRC37A4P*** | 2.59 | **0.000387903** | Up |
| ENSG00000288859 | ***H2AC19*** | 6.51 | **1.61791E-17** | Up |
| ENSG00000233937 | ***CTC-338M12.4*** | 2.58 | **1.45814E-06** | Up |
| ENSG00000279277 |  | 2.09 | **0.012605144** | Up |

**SMA_03**

| **ENS_ID** | ***Gene Symbol*** | **log2FC** | **FDR** | **SMA_T10 vs T0** |
| --- | --- | --- | --- | --- |
| ENSG00000179304 | ***FAM156B*** | -1.41 | **0.010114278** | Do |
| ENSG00000275895 |  | -6.99 | **2.19792E-35** | Do |
| ENSG00000101335 | ***MYL9*** | -1.70 | **0.000742458** | Do |
| ENSG00000186919 | ***ZACN*** | -4.36 | **6.28366E-15** | Do |
| ENSG00000203618 | ***GP1BB*** | -1.34 | **0.024261988** | Do |
| ENSG00000269145 | ***MAST3-AS1*** | -2.29 | **0.000126809** | Do |
| ENSG00000236304 |  | -1.75 | **0.000492729** | Do |
| ENSG00000005961 | ***ITGA2B*** | -2.24 | **9.47208E-09** | Do |
| ENSG00000154146 | ***NRGN*** | -1.27 | **0.022628885** | Do |
| ENSG00000113140 | ***SPARC*** | -1.43 | **0.00365196** | Do |
| ENSG00000280987 | ***MATR3*** | -5.91 | **7.33916E-19** | Do |
| ENSG00000248871 | ***TNFSF12-TNFSF13*** | -6.02 | **2.94762E-20** | Do |
| ENSG00000085733 | ***CTTN*** | -1.77 | **0.0422418** | Do |
| ENSG00000211892 | ***IGHG4*** | -4.33 | **1.64185E-41** | Do |
| ENSG00000163737 | ***PF4*** | -1.76 | **4.22455E-05** | Do |
| ENSG00000101162 | ***TUBB1*** | -1.35 | **0.009790108** | Do |
| ENSG00000258017 |  | -2.21 | **1.80478E-05** | Do |
| ENSG00000169704 | ***GP9*** | -1.87 | **0.00404601** | Do |
| ENSG00000285444 |  | -1.45 | **0.037289777** | Do |
| ENSG00000263020 |  | -1.90 | **0.000378629** | Do |
| ENSG00000103355 | ***PRSS33*** | -1.58 | **0.030910091** | Do |
| ENSG00000206047 | ***DEFA1*** | 3.01 | **1.08582E-17** | Up |
| ENSG00000224186 | ***PITX1-AS1*** | 3.40 | **3.59796E-11** | Up |
| ENSG00000268350 | ***FAM156A*** | 1.39 | **0.038174155** | Up |
| ENSG00000249624 |  | 2.22 | **1.33722E-06** | Up |
| ENSG00000227671 | ***ZNF731P*** | 1.48 | **0.009507064** | Up |
| ENSG00000257315 | ***ZBED6*** | 2.59 | **1.49655E-05** | Up |
| ENSG00000259332 | ***ST20-MTHFS*** | 2.28 | **0.001424103** | Up |
| ENSG00000257017 | ***HP*** | 1.59 | **0.007947976** | Up |
| ENSG00000096006 | ***CRISP3*** | 1.51 | **0.010213559** | Up |
| ENSG00000118113 | ***MMP8*** | 1.69 | **9.05493E-05** | Up |
| ENSG00000164821 | ***DEFA4*** | 1.38 | **0.037368202** | Up |
| ENSG00000164047 | ***CAMP*** | 1.35 | **0.016622733** | Up |

**SMA_05**

| **ENS_ID** | ***Gene Symbol*** | **log2FC** | **FDR** | **SMA_T10 vs T0** |
| --- | --- | --- | --- | --- |
| ENSG00000179304 | ***FAM156B*** | -7.06 | **3.83513E-21** | Do |
| ENSG00000275895 |  | -6.52 | **3.96021E-17** | Do |
| ENSG00000162366 | ***PDZK1IP1*** | -2.03 | **0.02671732** | Do |
| ENSG00000101335 | ***MYL9*** | -2.47 | **0.0001305** | Do |
| ENSG00000203618 | ***GP1BB*** | -1.99 | **0.025500868** | Do |
| ENSG00000236304 |  | -2.03 | **0.035098565** | Do |
| ENSG00000005961 | ***ITGA2B*** | -3.04 | **5.8822E-08** | Do |
| ENSG00000154146 | ***NRGN*** | -2.17 | **0.004130656** | Do |
| ENSG00000113140 | ***SPARC*** | -2.45 | **0.000110692** | Do |
| ENSG00000211895 | ***IGHA1*** | -1.95 | **0.049476786** | Do |
| ENSG00000184113 | ***CLDN5*** | -2.49 | **0.00251218** | Do |
| ENSG00000284874 |  | -2.27 | **0.003630067** | Do |
| ENSG00000211660 | ***IGLV2-23*** | -2.36 | **0.01127348** | Do |
| ENSG00000270550 | ***IGHV3-30*** | -2.29 | **0.013852751** | Do |
| ENSG00000267303 |  | -2.70 | **3.26728E-05** | Do |
| ENSG00000257607 |  | -2.34 | **0.00594472** | Do |
| ENSG00000170271 | ***FAXDC2*** | -2.36 | **0.001918188** | Do |
| ENSG00000085733 | ***CTTN*** | -2.39 | **0.003081173** | Do |
| ENSG00000173210 | ***ABLIM3*** | -2.44 | **0.004604436** | Do |
| ENSG00000149131 | ***SERPING1*** | -2.20 | **0.005489064** | Do |
| ENSG00000289100 |  | -2.29 | **0.000853087** | Do |
| ENSG00000082781 | ***ITGB5*** | -2.41 | **0.000964831** | Do |
| ENSG00000283633 |  | -2.23 | **0.004183936** | Do |
| ENSG00000108309 | ***RUNDC3A*** | -2.35 | **0.000670108** | Do |
| ENSG00000204420 | ***MPIG6B*** | -2.33 | **0.000880783** | Do |
| ENSG00000079308 | ***TNS1*** | -2.18 | **0.004293295** | Do |
| ENSG00000259207 | ***ITGB3*** | -2.39 | **0.000343115** | Do |
| ENSG00000166947 | ***EPB42*** | -1.97 | **0.038274633** | Do |
| ENSG00000184792 | ***OSBP2*** | -2.26 | **0.001583915** | Do |
| ENSG00000211896 | ***IGHG1*** | -2.55 | **3.8668E-05** | Do |
| ENSG00000137198 | ***GMPR*** | -1.99 | **0.028884718** | Do |
| ENSG00000120885 | ***CLU*** | -2.23 | **0.001583193** | Do |
| ENSG00000101162 | ***TUBB1*** | -2.04 | **0.013958567** | Do |
| ENSG00000162722 | ***TRIM58*** | -2.33 | **0.000532357** | Do |
| ENSG00000171552 | ***BCL2L1*** | -1.98 | **0.038869007** | Do |
| ENSG00000244437 | ***IGKV3-15*** | -2.41 | **0.020081913** | Do |
| ENSG00000211653 | ***IGLV1-40*** | -2.46 | **0.006266883** | Do |
| ENSG00000251546 | ***IGKV1D-39*** | -2.24 | **0.006296765** | Do |
| ENSG00000198892 | ***SHISA4*** | -2.82 | **0.000291612** | Do |
| ENSG00000278196 | ***IGLV2-8*** | -2.31 | **0.013507727** | Do |
| ENSG00000289316 |  | -2.58 | **0.000507559** | Do |
| ENSG00000070182 | ***SPTB*** | -2.06 | **0.034660285** | Do |
| ENSG00000029534 | ***ANK1*** | -2.16 | **0.010409562** | Do |
| ENSG00000285238 |  | 5.75 | **3.21203E-08** | Up |

**SMA_06**

| **ENS_ID** | ***Gene Symbol*** | **log2FC** | **FDR** | **SMA_T10 vs T0** |
| --- | --- | --- | --- | --- |
| ENSG00000206047 | ***DEFA1*** | -6.64 | **3.56874E-32** | Do |
| ENSG00000286190 |  | -2.83 | **4.53427E-14** | Do |
| ENSG00000285171 |  | -1.86 | **0.000408707** | Do |
| ENSG00000213999 | ***MEF2B*** | -4.17 | **3.14649E-18** | Do |
| ENSG00000211890 | ***IGHA2*** | -1.18 | **0.016554747** | Do |
| ENSG00000186919 | ***ZACN*** | 1.71 | **0.033216451** | Up |
| ENSG00000263244 |  | 1.83 | **1.11626E-06** | Up |
| ENSG00000254996 | ***ANKHD1-EIF4EBP3*** | 1.41 | **0.000434515** | Up |
| ENSG00000287542 | ***HERC3*** | 2.41 | **7.55873E-06** | Up |
| ENSG00000273047 |  | 2.13 | **9.50159E-05** | Up |

**SMA_09**

| **ENS_ID** | ***Gene Symbol*** | **log2FC** | **FDR** | **SMA_T10 vs T0** |
| --- | --- | --- | --- | --- |
| ENSG00000261796 | ***ISY1-RAB43*** | -1.36 | **0.00733129** | Do |
| ENSG00000278599 | ***TBC1D3E*** | -1.78 | **0.005603887** | Do |
| ENSG00000179304 | ***FAM156B*** | 5.93 | **1.76273E-21** | Up |
| ENSG00000205609 | ***EIF3CL*** | 1.56 | **0.01423154** | Up |
| ENSG00000269711 |  | 1.76 | **0.000287983** | Up |
| ENSG00000234857 | ***HNRNPUL2-BSCL2*** | 1.75 | **1.98689E-06** | Up |
